# Effects of Water, Sanitation, Handwashing, and Nutritional Interventions on Child Enteric Protozoan Infections in Rural Bangladesh: A Cluster-Randomized Controlled Trial

**DOI:** 10.1093/cid/ciy320

**Published:** 2018-04-13

**Authors:** Audrie Lin, Ayse Ercumen, Jade Benjamin-Chung, Benjamin F Arnold, Shimul Das, Rashidul Haque, Sania Ashraf, Sarker M Parvez, Leanne Unicomb, Mahbubur Rahman, Alan E Hubbard, Christine P Stewart, John M Colford, Stephen P Luby

**Affiliations:** 1 Division of Epidemiology and Biostatistics, School of Public Health, University of California, Berkeley; 2 Enteric and Respiratory Infections Programme, Infectious Diseases Division, International Centre for Diarrhoeal Disease Research, Bangladesh, Dhaka; 3 Department of Nutrition, University of California, Davis; 4 Infectious Diseases and Geographic Medicine, Stanford University, California

**Keywords:** water, sanitation, hygiene, nutrition, *Giardia*

## Abstract

**Background:**

We evaluated effects of individual and combined water, sanitation, handwashing (WSH), and nutritional interventions on protozoan infections in children.

**Methods:**

We randomized geographical clusters of pregnant women in rural Bangladesh into chlorinated drinking water, hygienic sanitation, handwashing, nutrition, combined WSH, nutrition plus WSH (N+WSH), or control arms. Participants were not masked. After approximately 2.5 years of intervention, we measured *Giardia*, *Cryptosporidium*, and *Entamoeba histolytica* prevalence and infection intensity by multiplex real-time polymerase chain reaction of child stool. Analysis was intention-to-treat.

**Results:**

Between May 2012 and July 2013, we randomized 5551 pregnant women. At follow-up, among 4102 available women, we enrolled 6694 children into the protozoan assessment. We analyzed stool from 5933 children (aged ~31 months) for protozoan infections. Compared with 35.5% prevalence among controls, *Giardia* infection prevalence was lower in the sanitation (26.5%; prevalence ratio [PR], 0.75 [95% confidence interval {CI}, .64–.88]), handwashing (28.2%; PR, 0.80 [95% CI, .66–.96]), WSH (29.7%; PR, 0.83 [95% CI, .72–.96]), and N+WSH (26.7%; PR, 0.75 [95% CI, .64–.88]) arms. Water and nutrition interventions had no effect. Low prevalence of *E. histolytica* and *Cryptosporidium* (<2%) resulted in imprecise effect estimates.

**Conclusions:**

Individual handwashing and hygienic sanitation interventions significantly reduced childhood *Giardia* infections, and there were no effects of chlorinated drinking water and nutrition improvements in this context. Combined WSH interventions provided no additional benefit. To reduce *Giardia* infection, individual WSH interventions may be more feasible and cost-effective than combined interventions in similar rural, low-income settings.

**Clinical Trials Registration:**

NCT01590095.

## Teaser

The trial found that individual handwashing and sanitation interventions significantly reduced childhood *Giardia* infections. Combined interventions provided no additional benefit. To reduce *Giardia* infection, individual interventions may be more feasible and cost-effective than combined interventions in similar rural, low-income settings.

In low-income countries, infections with enteric protozoan parasites have been associated with morbidity, malnutrition, and mortality [1, 2]. Global reduction of such infections would aid progress toward achieving Sustainable Development Goals for child health and survival (goal 3) [3]. Transmission of *Giardia duodenalis*, *Entamoeba histolytica*, and *Cryptosporidium* species is typically through the fecal–oral route [4–6]. Thus, children living in environments contaminated with feces have a higher risk of acquiring intestinal protozoan infections. There is limited and mixed evidence assessing the effect of combined water, sanitation, and handwashing (WSH), or nutritional interventions on protozoan infections: 2 trials with limited statistical power reported no effect of water treatment on *Cryptosporidium* or *Giardia* [7, 8], 1 water treatment trial reported a reduction in *Cryptosporidium* [9], 1 sanitation trial reported a reduction in *Giardia* [10], and exclusive breastfeeding and improved nutritional status potentially confer immunity against protozoan infections [11–13].

Because combined interventions are more expensive and difficult to implement than individual interventions, a longstanding question for policymakers within the WSH sector is whether combined interventions are more effective than individual interventions. We conducted a randomized controlled trial in rural Bangladesh to assess the impact of individual and combined water, sanitation, handwashing, and nutritional interventions on child health. All intervention arms except water treatment significantly reduced caregiver-reported diarrhea, and combining WSH interventions provided no additive benefit [14]. However, caregiver-reported diarrhea is susceptible to differential courtesy bias in nonblinded studies [15]. Here, we report intervention effects on infections by 3 common parasitic causes of diarrhea (*Giardia*, *Cryptosporidium*, and *E. histolytica*), objective, prespecified additional outcomes of the trial not influenced by potential reporting bias [5, 6, 16, 17].

## METHODS

### Study Design

We conducted the cluster-randomized WASH Benefits Bangladesh trial in the rural Gazipur, Mymensingh, Tangail, and Kishoreganj districts. The study design and rationale were previously published (see CONSORT [Consolidated Standards of Reporting Trials] checklist in the Supplementary Materials) [17]. Study protocols were approved by human subjects committees at International Centre for Diarrhoeal Disease Research, Bangladesh (icddr,b), the University of California, Berkeley, and Stanford University.

### Randomization

Eight neighboring compounds with eligible pregnant women formed a cluster. Clusters were separated by a minimum 1-km buffer to prevent spillover between clusters. Eight adjacent clusters formed a geographically matched randomization block. A University of California, Berkeley investigator (B. F. A.) used a random number generator to randomize matched clusters to the double-sized control arm or 1 of the 6 intervention arms (water; sanitation; handwashing; combined WSH; nutrition; or combined WSH plus nutrition [N+WSH]). Masking and sample size details are provided in the Supplementary Materials.

### Study Participants

We enrolled pregnant women who reported being in their first or second trimester of pregnancy. Children born to enrolled women were considered index children, and the household the index child lived in was considered the index household. Compounds in rural Bangladesh include a collection of households of extended families (3–4 households/compound) with a shared courtyard. Protozoan parasite outcomes in fecal samples were measured at enrollment and approximately 2.5 years after the beginning of intervention implementation. At enrollment, we assessed children living in the compound aged 18–27 months; their infection status provided baseline infection prevalence among children who were in the age range the birth cohort would be at the trial’s endpoint. After approximately 2.5 years of intervention, we tested samples as follows: (1) all index children (mean age 30 months at follow-up); (2) 1 child living in the enrolled compound aged 18–27 months at enrollment (same children measured at enrollment, aged 42–51 months at follow-up); and (3) 1 older child living in the enrolled compound aged 5–12 years at follow-up (we preferentially selected the index child’s sibling, followed by a child living in the same household as the index child, or same compound as the index child). School-aged children may have different intestinal protozoa transmission patterns compared to younger children. Primary caregivers of children provided written informed consent. Children aged 7–12 years provided written assent. Additional enrollment criteria are shown in the Supplementary Materials.

### Procedures

The interventions were previously described [14]. Interventions targeted the index child, index household, or compound containing the index household and included (1) chlorine-treated drinking water and safe storage vessel with spigot delivered to index households; (2) child potties and sani-scoop hoes delivered to index households to dispose of feces, and upgrades to double-pit latrines with hygienic water seals for all households in the compound; (3) handwashing stations with soapy water near the latrine and kitchen, delivered to the index households; (4) exclusive breastfeeding promotion (<6 months), lipid-based nutrient supplements (6–24 months), and age-appropriate maternal and infant nutrition recommendations (pregnancy to 24 months), all targeted to the index child; (5) combined WSH; and (6) N+WSH. Trained local women served as community health promoters (Supplementary Material) [14]. Promoters did not visit the control arm. Intervention adherence was high (>80%) for all interventions throughout the trial (Parvez et al, unpublished data).

### Outcomes

In the trial, protozoan parasite infections were prespecified tertiary outcomes [17], including the prevalence of *Giardia*, *Cryptosporidium*, and *E. histolytica* infections, infection with any of the 3 organisms, coinfection with 2 or 3 of the organisms, and intensity of each organism-specific infection (measured in cycle threshold [Ct] values by multiplex real-time polymerase chain reaction [18]; details in Supplementary Materials).

### Statistical Analysis

The preregistered analysis protocol (https://osf.io/2dtjk/) and full replication files are available (https://osf.io/c7u8b/). Analyses were conducted using R statistical software version 3.4.0.

Primary analysis included all children living in index households because this subset of children would most likely benefit from the household- and child-level interventions (handwashing, water treatment, and nutrition). Two secondary analyses included (1) index children who were primary recipients of the interventions; (2) all children in study compounds with protozoan parasite infection measured (including nonindex households).

Analyses were intention-to-treat. First, we compared each intervention arm against the double-sized control arm. Second, we assessed whether outcomes differed between combined WSH and the individual water, sanitation, and handwashing arms. Third, we tested whether outcomes differed between combined N+WSH and the individual nutrition or combined WSH arms.

The preregistered analytic approach for these analyses followed the same methods as the main trial [17]. Randomization led to highly balanced enrollment characteristics across arms, so we relied on the unadjusted analysis as our primary analysis. We estimated unadjusted prevalence ratio (PR), prevalence difference, and relative reduction in infection intensity (defined as the ratio of Ct values between arms minus 1) parameters using targeted maximum likelihood estimation (TMLE) [19]. For infection intensity analysis, a Ct value of 40 was imputed for samples classified as nondetects (reactions failing to pass the threshold level of minimum signal intensity) [20]. A secondary analysis adjusted for enrollment covariates associated with the outcome (likelihood ratio test *P* < .20) is detailed in the Supplementary Materials. We used inverse probability of censoring weighting with TMLE to correct for potential bias due to informative censoring (details in Supplementary Materials) [21].

### Trial Registration

The trial was registered at ClinicalTrials.gov (NCT01590095) in April 2012 and includes the trial’s primary and secondary outcomes (diarrhea and child growth). The study design was published in June 2013 and includes protozoa under tertiary outcomes [17]. The prespecified analysis plan for the protozoan outcomes was registered at Open Science Framework (https://osf.io/2dtjk/) in May 2017 before analysts had access to blinded protozoan outcome data.

## RESULTS

**Figure 1.**
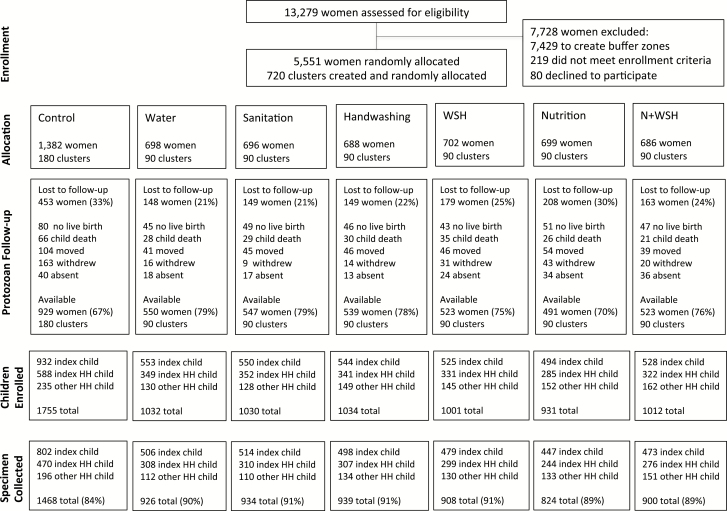
Flowchart of study participants’ progress through the phases of the trial. Index child is a child born to an enrolled pregnant woman, including twins. Index household refers to a household where an index child in the birth cohort lived. Index household child is an older child living in the index household who is not the index child. Other household child is a child who does not live in the index household but lives in the shared compound. Abbreviations: HH, household; N+WSH, combined nutrition, water treatment, sanitation, and handwashing.

Study staff identified 13279 pregnant women in their first or second trimester; 5551 (in 720 clusters) were randomly allocated to 1 of the intervention arms or the control between 31 May 2012 and 7 July 2013 (Figure 1). At the time of the protozoan parasite measurement between May 2015 and May 2016, 26% (n = 1449) of women were lost to follow-up. Reasons for loss to follow-up included no live birth (n = 361), index child death (n = 235), relocation (n = 375), withdrawal (n = 296), and absence (n = 182) (Figure 1). Controls had higher attrition (33%) compared with the intervention arms combined (24%) due to more withdrawals (12% vs 3%). Among 4102 available women, a total of 6694 children living in index households were enrolled in the protozoan parasite follow-up, and outcomes were measured in 5933 children (89%) with available stool specimens (Figure 1). Household enrollment characteristics were balanced across arms at follow-up (Table 1) and between children with parasite infections measured vs those with missing specimens (Supplementary Table 1).

**Table 1.** Enrollment Characteristics, by Intervention Group

Characteristic	Control	Water	Sanitation	Handwashing	WSH	Nutrition	N+WSH
No. of women	(n = 929)	(n = 550)	(n = 547)	(n = 539)	(n = 523)	(n = 491)	(n = 523)
Mothers							
Age, y, mean (range)	24 (15–43)	24 (15–43)	24 (15–41)	24 (15–60)	25 (15–44)	24 (15–45)	24 (14–43)
Years of education, mean (range)	6 (0–15)	6 (0–14)	6 (0–17)	6 (0–16)	6 (0–14)	6 (0–16)	6 (0–14)
Fathers							
Years of education, mean (range)	5 (0–16)	5 (0–16)	5 (0–17)	5 (0–16)	5 (0–16)	5 (0–16)	5 (0–16)
Works in agriculture, % (No.)	31 (292)	31 (173)	31 (168)	37 (202)	31 (162)	34 (165)	31 (163)
Household							
No. of persons, mean (range)	5 (2–17)	5 (2–23)	5 (2–17)	5 (2–22)	5 (1–14)	5 (2–18)	5 (2–14)
Has electricity, % (No.)	58 (538)	63 (345)	61 (331)	60 (322)	63 (330)	61 (301)	61 (317)
Has a cement floor, % (No.)	10 (93)	12 (66)	12 (66)	8 (43)	11 (56)	9 (42)	12 (63)
Acres of agricultural land owned, mean (range)	0.1 (0.0–2.5)	0.1 (0.0–2.4)	0.1 (0.0–3.2)	0.1 (0.0–2.6)	0.2 (0.0–3.1)	0.2 (0.0–2.8)	0.2 (0.0–8.9)
Drinking water, % (No.)							
Shallow tubewell primary water source	77 (711)	73 (404)	75 (411)	70 (379)	79 (413)	75 (369)	74 (387)
Stored water observed at home	47 (433)	51 (281)	47 (259)	49 (263)	41 (217)	42 (205)	48 (251)
Reported treating water yesterday	0 (3)	0 (1)	0 (0)	0 (1)	0 (0)	0 (0)	0 (2)
Sanitation							
Daily defecating in the open, % (No.)							
Adult men	7 (67)	5 (29)	7 (36)	10 (53)	7 (34)	7 (36)	7 (39)
Adult women	5 (44)	3 (14)	4 (23)	5 (28)	4 (21)	5 (26)	4 (20)
Children 8–14 y (n = 1743)	10 (38)	10 (21)	9 (22)	15 (37)	8 (19)	8 (17)	9 (22)
Children 3–7 y (n = 2179)	40 (197)	36 (111)	37 (109)	38 (110)	35 (99)	35 (85)	36 (99)
Children 0–2 y (n = 848)	81 (157)	86 (89)	81 (86)	85 (100)	78 (92)	83 (85)	89 (93)
Latrine, % (No.)							
Owned	53 (496)	53 (291)	53 (292)	55 (294)	53 (277)	54 (266)	54 (283)
Concrete slab	90 (840)	93 (510)	88 (483)	90 (483)	90 (471)	90 (440)	90 (472)
Functional water seal	25 (235)	26 (145)	26 (142)	25 (137)	21 (110)	26 (130)	23 (118)
Visible stool on slab or floor	49 (451)	45 (247)	45 (245)	44 (236)	53 (275)	46 (227)	49 (258)
Owned a child’s potty	3 (32)	4 (21)	4 (21)	5 (27)	4 (19)	5 (26)	5 (25)
Human feces observed in an area, % (No.)							
House	9 (84)	10 (53)	8 (42)	11 (57)	7 (37)	7 (35)	7 (36)
Child’s play area	1 (13)	1 (6)	1 (5)	1 (6)	1 (4)	1 (3)	1 (6)
Handwashing, % (No.)							
Has within 6 steps of latrine							
Water	13 (119)	12 (66)	12 (65)	9 (46)	8 (43)	9 (42)	12 (61)
Soap	5 (51)	7 (38)	8 (41)	5 (26)	5 (24)	4 (22)	6 (31)
Has within 6 steps of kitchen							
Water	9 (79)	7 (36)	7 (40)	6 (31)	8 (43)	9 (45)	9 (46)
Soap	2 (22)	2 (12)	2 (11)	2 (11)	2 (11)	4 (19)	3 (17)
Parasite prevalence							
Children: 18–27 mo, % (No.)	(n = 160)	(n = 87)	(n = 73)	(n = 84)	(n = 104)	(n = 100)	(n = 97)
*Giardia duodenalis*	53 (85)	52 (45)	49 (36)	55 (46)	53 (55)	48 (48)	61 (59)
*Cryptosporidium* spp	3 (5)	7 (6)	1 (1)	6 (5)	3 (3)	3 (3)	1 (1)
*Entamoeba histolytica*	3 (4)	1 (1)	3 (2)	4 (3)	0 (0)	1 (1)	3 (3)

Abbreviations: N+WSH, combined nutrition, water treatment, sanitation, and handwashing; WSH, combined water treatment, sanitation, and handwashing.

**Figure 2.**
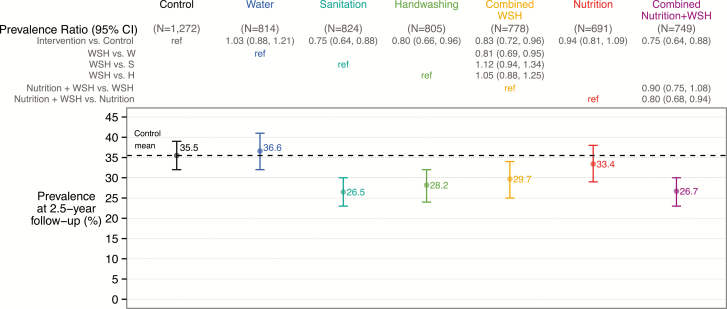
Intervention effects on *Giardia* prevalence among children living in index households approximately 2.5 years after intervention initiation. Abbreviations: CI, confidence interval; H, handwashing; S, sanitation; W, water treatment; WSH, combined water treatment, sanitation, and handwashing.

**Figure 3.**
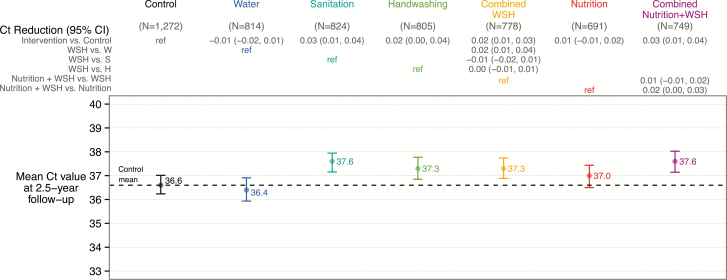
Intervention effects on *Giardia* infection intensity (cycle threshold [Ct] values) among children living in index households approximately 2.5 years after intervention initiation. Relative reduction of infection intensity is defined as CR – 1, where CR is the ratio of Ct values between arms. Nondetects were imputed as a Ct value of 40. Abbreviations: CI, confidence interval; Ct, cycle threshold; H, handwashing; S, sanitation; W, water treatment; WSH, combined water treatment, sanitation, and handwashing.

At trial enrollment, we measured protozoan parasites in stools from 705 children living in the compound aged 18–27 months to determine exposure prior to intervention initiation; 53.0%, 3.4%, and 2.0% were infected with *Giardia* species, *Cryptosporidium* species, and *E. histolytica*, respectively. Baseline protozoan infection prevalence was balanced across arms (Table 1). At follow-up, approximately 2.5 years after intervention initiation, the mean age was 29.9 (SD, 1.9) months for index children and 7.4 (SD, 1.9) years for nonindex children living in the index household. In the control group, 35.5%, 1.3%, and 0.3% of children living in index households were infected with *Giardia*, *Cryptosporidium*, and *E. histolytica*, respectively (Figure 2 and Supplementary Table 2). Among controls, the mean infection intensities as measured by Ct values were 36.6 (SD, 5.4) for *Giardia*, 39.9 (SD, 0.8) for *Cryptosporidium*, and 40.0 (SD, 0.6) for *E. histolytica* (Figure 3 and Supplementary Table 5).

Our prespecified primary analysis included all children living in index households. Compared to controls, *Giardia* infection prevalence was lower in the sanitation (26.5%; PR, 0.75 [95% confidence interval {CI}, .64–.88]), handwashing (28.2%; PR, 0.80 [95% CI, .66–.96]), combined WSH (29.7%; PR, 0.83 [95% CI, .72–.96]), and N+WSH arms (26.7%; PR, 0.75 [95% CI, .64–.88]) (Figure 1 and Supplementary Table 2). *Giardia* infection prevalence was not different in the individual water treatment arm (36.6%; PR, 1.03 [95% CI, .91–1.24]) and the nutrition arm (33.4%; PR, 0.94 [95% CI, .81–1.09]). Unadjusted, adjusted, and inverse probability of censoring weighting analyses yielded similar estimates (Supplementary Table 2). The low prevalence of *E. histolytica* and *Cryptosporidium* infections (<2% prevalence) resulted in highly imprecise intervention effect estimates for these parasites and coinfections with multiple parasites (Supplementary Table 2). Moreover, due to the low prevalence of *E. histolytica* and *Cryptosporidium*, the intervention effects on infection prevalence with any of the 3 protozoan parasites reiterated *Giardia* results. Combined WSH interventions did not reduce infection prevalence more than individual handwashing and sanitation interventions, and N+WSH did not reduce prevalence more than WSH (Figure 2 and Supplementary Tables 3 and 4). The *Giardia* infection intensity results mirrored the prevalence results (Figure 3 and Supplementary Tables 5–7).

Our 2 prespecified secondary analyses included index children (who were the primary recipients of the interventions) and all children living in study compounds with protozoan parasite infection measured (including nonindex households). Among index children, compared with 31.8% prevalence in the control group, *Giardia* infection prevalence was significantly reduced 6–10 percentage points by sanitation, WSH, and N+WSH interventions; however, unlike the index household results, the reduction in the handwashing group was not significant (Supplementary Table 8). Similar to the index household results, among index children, water treatment and nutrition had no effect (Supplementary Table 8), combined interventions were not more effective compared to individual interventions (Supplementary Tables 9 and 10), and infection intensity results reflected prevalence results (Supplementary Tables 11–13). The analysis including all children living in study compounds was similar to the index household primary results (Supplementary Tables 14–19). In an additional analysis among children in index households who were positive for infection (not prespecified), interventions had no effect on infection intensity (Supplementary Table 20).

## DISCUSSION

Our results show that individual handwashing and hygienic sanitation interventions significantly reduced childhood *Giardia* infections to a similar degree. Chlorinated drinking water treatment and nutrition interventions had no effect on *Giardia* infections. Furthermore, the combination of these WSH and N+WSH interventions did not provide additional benefits beyond individual handwashing and hygienic sanitation interventions.

We observed a secular decline in *Giardia* prevalence between children aged 18–27 months at enrollment prior to intervention initiation and similarly aged (~30 months) index children in the control arm at follow-up (53% vs 32% prevalence). This secular decline in *Giardia* prevalence is consistent with the main outcomes of the trial that reported lower prevalence of diarrhea at follow-up than has previously been reported among young children in rural Bangladesh [22]. Nevertheless, the randomized controlled trial design and balanced household enrollment characteristics, including balanced baseline protozoan prevalence across arms, enabled us to draw valid inferences about intervention effects in the presence of secular trends.

The main outcome trial reported significant reductions in caregiver-reported diarrhea in all of the intervention arms except water treatment and no additive effect of combined WSH interventions [14]; however, this subjective outcome could be susceptible to courtesy bias in an unblinded trial [15]. Although 2 of the largest studies of pediatric diarrhea etiology found that *Giardia* was not associated with diarrhea [23, 24], *Giardia* is a known enteropathogen among immunologically naive populations and may be associated with childhood growth faltering [5]. The *Giardia* results provide objective evidence of a reduction in enteropathogen transmission with handwashing and hygienic sanitation interventions. Furthermore, the *Giardia* reductions in prevalence and infection intensity in the individual sanitation and handwashing arms, but not water treatment arm, were consistent with the diarrhea results. The lack of additive benefit on *Giardia* from combining the WSH interventions further aligned with the diarrhea findings. The high levels of internal consistency between the effects on *Giardia* and caregiver-reported diarrhea lend additional credibility to the reported diarrhea results and suggest that this reported outcome was not affected by differential reporting bias. Taken together, these results provide evidence for the lack of additive benefit in combining WSH interventions on *Giardia* and diarrhea. These results may have policy implications in the context of similar rural settings: per dollar invested, implementing effective individual interventions to a larger population may prevent more childhood *Giardia* infections and diarrhea than allocating identical funding for combined interventions implemented among a smaller population.

The sanitation intervention consisting of compound-level hygienic double-pit latrines and household-level sani-scoop hoes and child potties along with behavior promotion reduced *Giardia* prevalence and infection intensity [25–27]. These sanitation interventions likely disrupted key fecal–oral transmission pathways via safe containment and disposal of feces in the compound and household living environments, thereby reducing exposure of children to *Giardia* [5]. Although our compound-level hygienic sanitation upgrades were different in coverage and scope, this result is consistent with a previous trial of a community-level sanitation program that aimed to end open defecation by changing behaviors in rural India [10]. The sanitation intervention in our trial achieved a significant 9 percentage point reduction in *Giardia* prevalence from 35.5% prevalence among controls. The magnitude of this reduction was larger than the borderline significant 5 percentage point reduction from 23.2% prevalence among controls in the India trial [10]. These differences in reduction may be attributed to the high intervention adherence achieved in this trial compared to the relatively low adherence observed in the India trial.

The household-level provision of handwashing stations with soapy water near the latrine and kitchen, accompanied by handwashing promotion, significantly lowered *Giardia* prevalence and infection intensity [28]. The handwashing intervention may have limited *Giardia* transmission via caregivers’ hands and contaminated food, common routes of transmission [5].

Consistent with in vitro studies demonstrating that *Giardia* is a chlorine-resistant pathogen [29], the household-level chlorine-based water treatment and safe storage intervention in our trial had no impact on childhood *Giardia* infection. Boiling and filtration-based water treatment interventions may be more effective at inactivating or removing *Giardia* cysts than chlorination [30].

The nutrition intervention had no effect on *Giardia* infections among index children aged approximately 30 months, the primary recipients of the intervention. A multisite birth-cohort study identified exclusive breastfeeding and better nutritional status as likely protective factors against subsequent *Giardia* infections [12]. By age 30 months, the combination of incomplete adherence to exclusive breastfeeding, waning breastfeeding practices, and repeated *Giardia* infections resulting from contaminated food and water could contribute to the null effect of the nutrition intervention. Studies have reported associations between *Giardia* infections before 6 months of age and subsequent linear growth deficits [12, 31]. Future analyses of banked specimens collected from study children prior to 30 months could provide insight on potential early intervention effects in the nutrition arm.

This study had limitations. First, these results from a rural, low-income setting in Bangladesh during a time of unusually low diarrhea prevalence may not generalize to other settings or time periods. Second, we did not determine *Giardia* genotype, a potential factor that may contribute to heterogeneity in the clinical manifestation of infections [32]. Third, this study did not measure protozoan infection status after intervention initiation but before the age of 2 years, preventing inference on potential early intervention effects. Persistent *Giardia* infections during sensitive windows of development among young children may adversely impact growth [12]. Finally, cases of *Cryptosporidium* and *E. histolytica* infections were sufficiently rare in the population that we could not measure the effects of the interventions with precision.

In summary, interventions that combined WSH components or added nutrition provided no additional benefit for *Giardia* infections beyond individual handwashing and hygienic sanitation interventions. One possible explanation for the lack of additive benefit is differential adherence to interventions. It is possible that more complex combined interventions require more substantial behavior change and, therefore, achieve lower uptake [33]. However, sanitation uptake was similar across single and combined intervention arms in our study; handwashing uptake in the individual arm was only slightly higher than in the combined WSH arms (93%–94% vs 85%–87% of households had water and soap at handwashing stations near the kitchen and latrine; *P* < .01) (Parvez et al, unpublished data). Alternatively, *Giardia* could be transmitted through interdependent pathways in this setting [34], and individual sanitation and handwashing interventions could be interrupting the same transmission pathway. Due to this potential redundancy, either intervention might be sufficient to reduce *Giardia* infection, but a combination of the interventions would produce no additive benefit [35]. From a cost-effectiveness perspective, each intervention package included similar behavior change promotion efforts, but the hardware costs for handwashing stations with soapy water were lower than for latrine construction. Because the 2 interventions reduced *Giardia* prevalence by similar amounts, handwashing interventions could be a more cost-effective strategy than latrine construction to reduce *Giardia* infection in this setting. Evaluating this strategy within other low-income contexts with high diarrheal prevalence and utilizing a broader array of enteropathogens could provide valuable insights to this sector.

## Supplementary Data

Supplementary materials are available at *Clinical Infectious Diseases* online. Consisting of data provided by the authors to benefit the reader, the posted materials are not copyedited and are the sole responsibility of the authors, so questions or comments should be addressed to the corresponding author.

Supplementary Tables

Supplementary Material

## Notes

***Acknowledgments.*** We greatly appreciate the families who participated in the study and the dedication of the icddr,b staff who delivered the interventions and collected the data and specimens. We thank Mohammad Zahidur Rahman and Mohammad Alimojjaman for supervising data and specimen collection.

***Financial support.*** This research was financially supported by the Bill & Melinda Gates Foundation (Global Development grant number OPPGD759 to the University of California, Berkeley). The icddr,b is grateful to the governments of Bangladesh, Canada, Sweden, and the United Kingdom for providing core/unrestricted support.

***Potential conflicts of interest.*** All authors: No potential conflicts. All authors have submitted the ICMJE Form for Disclosure of Potential Conflicts of Interest. Conflicts that the editors consider relevant to the content of the manuscript have been disclosed.
