# Supplementary material for: Effects of Water, Sanitation, Handwashing, and Nutritional Interventions on Child Enteric Protozoan Infections in Rural Bangladesh: A Cluster-Randomized Controlled Trial
Source: Clin Infect Dis. 2018 Apr 13;67(10):1515–22. doi: 10.1093/cid/ciy320 (PMC6206106; doi:10.1093/cid/ciy320)
Supplement: Supplementary Tables [file ciy320_suppl_supplementary_tables.pdf]

Table S1: Enrollment characteristics of individuals with missing vs. observed outcomes

No. of individuals:	Missing (N=3112)	Observed (N=6899)
Index child, %	48.3	53.9
**Maternal**		
Age, mean	23.2	24.3
Years of education, mean	5.8	5.7
**Paternal**		
Years of education, mean	4.9	4.7
Works in agriculture, %	28.3	33.2
**Household**		
Number of persons, mean	4.7	4.8
Has electricity, %	56.8	59.1
Has a cement floor, %	12.0	9.6
Acres of agricultural land owned, mean	0.1	0.1
**Drinking water**		
Shallow tubewell primary water source, %	72.1	74.0
Stored water observed at home, %	50.5	46.6
Reported treating water yesterday, %	0.0	0.2
**Sanitation**		
Daily defecating in the open, %		
Adult men	7.5	8.1
Adult women	3.9	4.9
Children: 8-<15 years	9.3	11.1
Children: 3-<8 years	36.4	38.1
Children: 0-<3 years	86.2	83.6
Latrine, %		
Owned	51.1	51.7
Concrete slab	90.3	88.8
Functional water seal	25.8	23.6
Visible stool on slab or floor	47.8	48.2
Owned a potty, %	5.5	4.5
Human feces observed in, %		
House	8.0	9.2
Child's play area	1.4	1.2
**Handwashing**		
Has within 6 steps of latrine, %		
Water	12.5	10.0
Soap	7.5	5.1
Has within 6 steps of kitchen, %		
Water	8.2	7.5
Soap	2.2	2.3

Table S2: Protozoa prevalence among index households approximately 2.5 years after intervention initiation, all interventions vs. control

Arm	N	Prev	Prevalence ratio			Prevalence difference		
			Unadjusted	Adjusted [a]	IPCW [b]	Unadjusted	Adjusted [a]	IPCW [b]
Giardia								
Control	1272	35.5%						
Water	814	36.6%	1.03 (0.88, 1.21)	1.06 (0.91, 1.24)	1.05 (0.90, 1.23)	1.14 (-4.46, 6.74)	2.12 (-3.47, 7.71)	1.86 (-3.71, 7.43)
Sanitation	824	26.5%	0.75 (0.64, 0.88)	0.75 (0.66, 0.87)	0.75 (0.65, 0.86)	-8.97 (-13.79, -4.15)	-8.63 (-12.88, -4.38)	-8.80 (-13.10, -4.50)
Handwashing	805	28.2%	0.80 (0.66, 0.96)	0.78 (0.66, 0.94)	0.78 (0.66, 0.93)	-7.26 (-12.91, -1.61)	-7.65 (-12.98, -2.33)	-7.77 (-13.02, -2.52)
WSH	778	29.7%	0.83 (0.72, 0.96)	0.84 (0.74, 0.97)	0.84 (0.73, 0.96)	-5.98 (-10.43, -1.53)	-5.53 (-9.90, -1.16)	-5.78 (-10.23, -1.33)
Nutrition	691	33.4%	0.94 (0.81, 1.09)	0.93 (0.80, 1.07)	0.93 (0.80, 1.08)	-1.99 (-7.07, 3.08)	-2.60 (-7.61, 2.40)	-2.41 (-7.39, 2.57)
Nutrition + WSH	749	26.7%	0.75 (0.64, 0.88)	0.78 (0.68, 0.91)	0.77 (0.67, 0.90)	-8.76 (-13.36, -4.15)	-7.57 (-11.92, -3.21)	-7.91 (-12.27, -3.54)
E. histolytica								
Control	1272	0.3%						
Water	814	0.4%	1.18 (0.23, 5.97)	1.15 (0.24, 5.53)	1.18 (0.23, 5.93)	0.06 (-0.52, 0.63)	0.05 (-0.50, 0.59)	0.06 (-0.52, 0.63)
Sanitation	824	0.5%	1.54 (0.31, 7.67)	1.48 (0.29, 7.44)	1.41 (0.27, 7.31)	0.17 (-0.47, 0.81)	0.16 (-0.50, 0.81)	0.13 (-0.53, 0.79)
Handwashing	805	1.1%	3.57 (0.83, 15.31)	3.43 (0.81, 14.60)	3.53 (0.81, 15.28)	0.81 (-0.17, 1.78)	0.78 (-0.19, 1.75)	0.81 (-0.21, 1.82)
WSH	778	0.0%	– [c]	– [c]	– [c]	-0.32 (-0.69, 0.06)	-0.30 (-0.65, 0.05)	-0.30 (-0.66, 0.06)
Nutrition	691	1.3%	4.16 (1.69, 10.24)	4.14 (1.67, 10.25)	3.96 (1.58, 9.95)	0.99 (0.11, 1.87)	1.01 (0.14, 1.89)	0.95 (0.10, 1.79)
Nutrition + WSH	749	0.3%	0.85 (0.11, 6.35)	0.86 (0.12, 6.30)	0.85 (0.13, 5.60)	-0.05 (-0.63, 0.54)	-0.05 (-0.63, 0.54)	-0.05 (-0.60, 0.50)
Cryptosporidium								
Control	1272	1.3%						
Water	814	0.7%	0.58 (0.13, 2.60)	0.54 (0.13, 2.30)	0.60 (0.14, 2.62)	-0.52 (-1.78, 0.73)	-0.61 (-1.79, 0.58)	-0.51 (-1.76, 0.75)
Sanitation	824	1.1%	0.87 (0.38, 2.00)	0.90 (0.40, 1.99)	0.92 (0.42, 2.02)	-0.17 (-1.13, 0.80)	-0.13 (-1.06, 0.80)	-0.10 (-1.03, 0.83)
Handwashing	805	1.4%	1.09 (0.48, 2.45)	1.18 (0.55, 2.54)	1.17 (0.55, 2.50)	0.11 (-0.95, 1.18)	0.22 (-0.80, 1.24)	0.21 (-0.79, 1.21)
WSH	778	0.9%	0.69 (0.21, 2.25)	0.77 (0.24, 2.45)	0.75 (0.24, 2.34)	-0.39 (-1.60, 0.82)	-0.29 (-1.54, 0.95)	-0.31 (-1.52, 0.90)
Nutrition	691	0.7%	0.58 (0.18, 1.87)	0.57 (0.20, 1.64)	0.62 (0.20, 1.85)	-0.53 (-1.65, 0.58)	-0.54 (-1.55, 0.47)	-0.47 (-1.53, 0.58)
Nutrition + WSH	749	1.2%	0.95 (0.40, 2.26)	1.08 (0.50, 2.35)	1.07 (0.48, 2.37)	-0.06 (-1.11, 1.00)	0.10 (-0.89, 1.08)	0.09 (-0.92, 1.09)
**Any protozoa**								
Control	1272	36.3%						
Water	814	37.5%	1.03 (0.88, 1.20)	1.06 (0.91, 1.23)	1.05 (0.90, 1.22)	1.14 (-4.54, 6.81)	2.06 (-3.57, 7.70)	1.88 (-3.76, 7.53)
Sanitation	824	27.5%	0.76 (0.65, 0.89)	0.77 (0.66, 0.88)	0.76 (0.66, 0.88)	-8.74 (-13.76, -3.72)	-8.43 (-12.89, -3.98)	-8.70 (-13.22, -4.18)
Handwashing	805	29.7%	0.82 (0.68, 0.98)	0.81 (0.68, 0.96)	0.80 (0.68, 0.95)	-6.63 (-12.36, -0.90)	-7.05 (-12.50, -1.59)	-7.13 (-12.51, -1.75)
WSH	778	30.6%	0.84 (0.73, 0.96)	0.85 (0.74, 0.98)	0.84 (0.73, 0.97)	-5.98 (-10.63, -1.33)	-5.50 (-10.13, -0.87)	-5.77 (-10.47, -1.06)
Nutrition	691	34.7%	0.96 (0.83, 1.11)	0.95 (0.82, 1.10)	0.95 (0.82, 1.09)	-1.55 (-6.73, 3.64)	-1.91 (-7.03, 3.22)	-1.94 (-7.01, 3.13)
Nutrition + WSH	749	27.5%	0.76 (0.65, 0.88)	0.79 (0.68, 0.91)	0.78 (0.67, 0.90)	-8.82 (-13.48, -4.16)	-7.54 (-11.93, -3.14)	-7.97 (-12.42, -3.52)
**Multiple protozoa**								
Control	1272	0.7%						
Water	814	0.1%	0.18 (0.00, 6.29)	0.18 (0.01, 5.69)	0.18 (0.01, 5.78)	-0.58 (-1.28, 0.11)	-0.57 (-1.24, 0.10)	-0.57 (-1.24, 0.10)
Sanitation	824	0.5%	0.68 (0.21, 2.20)	0.75 (0.26, 2.18)	0.74 (0.25, 2.17)	-0.22 (-0.90, 0.46)	-0.17 (-0.80, 0.46)	-0.18 (-0.80, 0.45)
Handwashing	805	1.0%	1.41 (0.45, 4.40)	1.47 (0.50, 4.29)	1.46 (0.50, 4.24)	0.29 (-0.68, 1.25)	0.32 (-0.60, 1.24)	0.32 (-0.59, 1.23)
WSH	778	0.0%	– [c]	– [c]	– [c]	-0.70 (-1.30, -0.11)	-0.69 (-1.25, -0.12)	-0.69 (-1.26, -0.12)
Nutrition	691	0.7%	1.02 (0.31, 3.37)	1.00 (0.34, 2.91)	1.04 (0.35, 3.09)	0.02 (-0.84, 0.87)	-0.00 (-0.77, 0.77)	0.03 (-0.76, 0.81)
Nutrition + WSH	749	0.7%	0.94 (0.30, 2.98)	1.12 (0.39, 3.19)	1.03 (0.36, 2.94)	-0.04 (-0.83, 0.75)	0.08 (-0.67, 0.83)	0.02 (-0.72, 0.76)

[a] See pre-specified analysis plan for adjustment covariates.
[b] Inverse probability of censoring weighting.
[c] Could not calculate due to sparse data.

Table S3: Protozoa prevalence among index households approximately 2.5 years after intervention initiation, combined vs. individual WSH

			Prevalence ratio			Prevalence difference		
Arm	N	Prev	Unadjusted	Adjusted [a]	IPCW [b]	Unadjusted	Adjusted [a]	IPCW [b]
Giardia								
WSH	778	29.7%						
Water	814	36.6%	0.81 (0.69, 0.95)	0.79 (0.68, 0.92)	0.79 (0.68, 0.92)	-6.90 (-12.05, -1.75)	-7.81 (-12.91, -2.72)	-7.77 (-12.78, -2.77)
Sanitation	824	26.5%	1.12 (0.94, 1.34)	1.13 (0.95, 1.33)	1.12 (0.95, 1.33)	3.28 (-1.75, 8.31)	3.33 (-1.46, 8.12)	3.31 (-1.50, 8.13)
Handwashing	805	28.2%	1.05 (0.88, 1.25)	1.08 (0.90, 1.28)	1.08 (0.90, 1.28)	1.48 (-3.54, 6.50)	2.12 (-2.92, 7.16)	2.09 (-2.93, 7.11)
E. histolytica								
WSH	778	0.0%						
Water	814	0.4%	– [c]	– [c]	– [c]	-0.37 (-0.84, 0.10)	-0.37 (-0.85, 0.11)	-0.37 (-0.85, 0.12)
Sanitation	824	0.5%	– [c]	– [c]	– [c]	-0.48 (-0.95, -0.02)	-0.48 (-0.94, -0.03)	-0.48 (-0.95, -0.02)
Handwashing	805	1.1%	– [c]	– [c]	– [c]	-1.12 (-1.99, -0.25)	-1.12 (-2.07, -0.18)	-1.12 (-2.08, -0.17)
Cryptosporidium								
WSH	778	0.9%						
Water	814	0.7%	1.22 (0.49, 3.05)	1.27 (0.53, 3.03)	1.20 (0.50, 2.86)	0.16 (-0.59, 0.92)	0.20 (-0.54, 0.94)	0.15 (-0.58, 0.88)
Sanitation	824	1.1%	0.83 (0.29, 2.35)	0.77 (0.27, 2.18)	0.82 (0.30, 2.22)	-0.19 (-1.20, 0.83)	-0.26 (-1.27, 0.75)	-0.20 (-1.18, 0.78)
Handwashing	805	1.4%	0.66 (0.23, 1.92)	0.68 (0.23, 2.02)	0.68 (0.24, 1.94)	-0.46 (-1.62, 0.70)	-0.43 (-1.64, 0.77)	-0.44 (-1.61, 0.73)
**Any protozoa**								
WSH	778	30.6%						
Water	814	37.5%	0.82 (0.70, 0.95)	0.80 (0.68, 0.93)	0.80 (0.69, 0.92)	-6.86 (-12.04, -1.68)	-7.78 (-12.91, -2.64)	-7.77 (-12.82, -2.73)
Sanitation	824	27.5%	1.11 (0.93, 1.33)	1.11 (0.94, 1.31)	1.11 (0.94, 1.32)	3.09 (-2.04, 8.23)	3.06 (-1.79, 7.92)	3.12 (-1.78, 8.01)
Handwashing	805	29.7%	1.03 (0.87, 1.22)	1.05 (0.89, 1.25)	1.05 (0.88, 1.25)	0.89 (-4.24, 6.02)	1.53 (-3.65, 6.71)	1.43 (-3.74, 6.61)
**Multiple protozoa**								
WSH	778	0.0%						
Water	814	0.1%	– [c]	– [c]	– [c]	-0.12 (-0.34, 0.10)	-0.11 (-0.34, 0.12)	-0.11 (-0.34, 0.12)
Sanitation	824	0.5%	– [c]	– [c]	– [c]	-0.49 (-0.97, -0.01)	-0.50 (-0.95, -0.04)	-0.50 (-0.95, -0.04)
Handwashing	805	1.0%	– [c]	– [c]	– [c]	-0.99 (-1.68, -0.30)	-1.00 (-1.72, -0.28)	-1.00 (-1.72, -0.28)

[a] See pre-specified analysis plan for adjustment covariates.
[b] Inverse probability of censoring weighting.
[c] Could not calculate due to sparse data.

Table S4: Protozoa prevalence among index households approximately 2.5 years after intervention initiation, combined nutrition plus WSH vs. WSH and nutrition

			Prevalence ratio			Prevalence difference		
Arm	N	Prev	Unadjusted	Adjusted [a]	IPCW [b]	Unadjusted	Adjusted [a]	IPCW [b]
Giardia								
Nutrition + WSH	749	26.7%						
WSH	778	29.7%	0.90 (0.75, 1.08)	0.91 (0.76, 1.09)	0.92 (0.77, 1.10)	-2.99 (-8.14, 2.16)	-2.64 (-7.69, 2.41)	-2.35 (-7.39, 2.69)
Nutrition	691	33.4%	0.80 (0.68, 0.94)	0.82 (0.70, 0.96)	0.82 (0.70, 0.96)	-6.70 (-11.45, -1.96)	-5.81 (-10.43, -1.20)	-5.97 (-10.57, -1.36)
E. histolytica								
Nutrition + WSH	749	0.3%						
WSH	778	0.0%	– [c]	– [c]	– [c]	0.27 (-0.07, 0.61)	0.29 (-0.04, 0.61)	0.27 (-0.06, 0.60)
Nutrition	691	1.3%	0.21 (0.04, 1.02)	0.23 (0.05, 1.07)	0.21 (0.04, 1.01)	-1.04 (-2.33, 0.26)	-0.96 (-2.20, 0.28)	-1.01 (-2.24, 0.22)
Cryotosporidium								
Nutrition + WSH	749	1.2%						
WSH	778	0.9%	1.34 (0.42, 4.22)	1.31 (0.43, 4.06)	1.36 (0.45, 4.09)	0.30 (-0.89, 1.49)	0.29 (-0.89, 1.47)	0.32 (-0.83, 1.47)
Nutrition	691	0.7%	1.66 (0.49, 5.56)	1.73 (0.51, 5.84)	1.65 (0.47, 5.76)	0.48 (-0.60, 1.56)	0.52 (-0.57, 1.61)	0.47 (-0.65, 1.60)
**Any protozoa**								
Nutrition + WSH	749	27.5%						
WSH	778	30.6%	0.90 (0.76, 1.07)	0.91 (0.77, 1.08)	0.92 (0.78, 1.09)	-3.09 (-8.11, 1.92)	-2.79 (-7.75, 2.17)	-2.44 (-7.38, 2.51)
Nutrition	691	34.7%	0.79 (0.68, 0.92)	0.81 (0.70, 0.95)	0.81 (0.70, 0.94)	-7.20 (-11.89, -2.51)	-6.34 (-10.91, -1.76)	-6.51 (-11.10, -1.92)
**Multiple protozoa**								
Nutrition + WSH	749	0.7%						
WSH	778	0.0%	– [c]	– [c]	– [c]	0.67 (0.07, 1.27)	0.73 (0.13, 1.32)	0.69 (0.10, 1.29)
Nutrition	691	0.7%	0.91 (0.26, 3.23)	1.01 (0.29, 3.51)	0.89 (0.25, 3.16)	-0.06 (-0.94, 0.82)	0.01 (-0.86, 0.88)	-0.08 (-0.97, 0.81)

[a] See pre-specified analysis plan for adjustment covariates.
[b] Inverse probability of censoring weighting.
[c] Could not calculate due to sparse data.

Table S5: Ct reduction [a] among index households approximately 2.5 years after intervention initiation, all interventions vs. control

Arm	N	Mean	Unadjusted	Adjusted [b]	IPCW [c]
Giardia					
Control	1272	36.6			
Water	814	36.4	-0.01 (-0.02, 0.01)	-0.01 (-0.02, 0.01)	-0.01 (-0.02, 0.01)
Sanitation	824	37.6	0.03 (0.01, 0.04)	0.02 (0.01, 0.04)	0.02 (0.01, 0.04)
Handwashing	805	37.3	0.02 (0.00, 0.04)	0.02 (0.00, 0.04)	0.02 (0.00, 0.04)
WSH	778	37.3	0.02 (0.01, 0.03)	0.02 (0.01, 0.03)	0.02 (0.01, 0.03)
Nutrition	691	37.0	0.01 (-0.01, 0.02)	0.01 (-0.01, 0.02)	0.01 (-0.01, 0.02)
Nutrition + WSH	749	37.6	0.03 (0.01, 0.04)	0.02 (0.01, 0.04)	0.02 (0.01, 0.04)
E. histolytica [d]					
Control	1272	40.0			
Water	814	40.0	–	–	–
Sanitation	824	40.0	–	–	–
Handwashing	805	39.9	–	–	–
WSH	778	40.0	–	–	–
Nutrition	691	39.9	–	–	–
Nutrition + WSH	749	40.0	–	–	–
Cryptosporidium [d]					
Control	1272	39.9			
Water	814	39.9	–	–	–
Sanitation	824	39.9	–	–	–
Handwashing	805	39.9	–	–	–
WSH	778	39.9	–	–	–
Nutrition	691	40.0	–	–	–
Nutrition + WSH	749	39.9	–	–	–

[a] Ct reduction defined as Ct ratio (CtR) - 1, where CtR is the ratio of Ct values between arms.
[b] See pre-specified analysis plan for adjustment covariates.
[c] Inverse probability of censoring weighting.
[d] Could not calculate due to sparse data.

Table S6: Ct reduction [a] among index households approximately 2.5 years after intervention initiation, combined vs. individual WSH

Arm	N	Mean	Unadjusted	Adjusted [b]	IPCW [c]
Giardia					
WSH	778	37.3			
Water	814	36.4	0.02 (0.01, 0.04)	0.03 (0.01, 0.04)	0.03 (0.01, 0.04)
Sanitation	824	37.6	-0.01 (-0.02, 0.01)	-0.01 (-0.02, 0.01)	-0.01 (-0.02, 0.01)
Handwashing	805	37.3	0.00 (-0.01, 0.01)	-0.00 (-0.02, 0.01)	-0.00 (-0.01, 0.01)
E. histolytica [d]					
WSH	778	40.0			
Water	814	40.0	–	–	–
Sanitation	824	40.0	–	–	–
Handwashing	805	39.9	–	–	–
Cryptosporidium [d]					
WSH	778	39.9			
Water	814	39.9	–	–	–
Sanitation	824	39.9	–	–	–
Handwashing	805	39.9	–	–	–

[a] Ct reduction defined as Ct ratio (CtR) - 1, where CtR is the ratio of Ct values between arms.
[b] See pre-specified analysis plan for adjustment covariates.
[c] Inverse probability of censoring weighting.
[d] Could not calculate due to sparse data.

Table S7: Ct reduction [a] among index households approximately 2.5 years after intervention initiation, combined nutrition plus WSH vs. WSH and nutrition

Arm	N	Mean	Unadjusted	Adjusted [b]	IPCW [c]
Giardia					
Nutrition + WSH	749	37.6			
WSH	778	37.3	0.01 (-0.01, 0.02)	0.01 (-0.01, 0.02)	0.01 (-0.01, 0.02)
Nutrition	691	37.0	0.02 (0.00, 0.03)	0.01 (0.00, 0.03)	0.01 (0.00, 0.03)
E. histolytica [d]					
Nutrition + WSH	749	40.0			
WSH	778	40.0	–	–	–
Nutrition	691	39.9	–	–	–
Cryptosporidium [d]					
Nutrition + WSH	749	39.9			
WSH	778	39.9	–	–	–
Nutrition	691	40.0	–	–	–

[a] Ct reduction defined as Ct ratio (CtR) - 1, where CtR is the ratio of Ct values between arms.
[b] See pre-specified analysis plan for adjustment covariates.
[c] Inverse probability of censoring weighting.
[d] Could not calculate due to sparse data.

Table S8: Protozoa prevalence among index children approximately 2.5 years after intervention initiation, all interventions vs. control

			Prevalence ratio			Prevalence difference		
Arm	N	Prev	Unadjusted	Adjusted [a]	IPCW [b]	Unadjusted	Adjusted [a]	IPCW [b]
Giardia								
Control	802	31.8%						
Water	506	34.2%	1.07 (0.90, 1.29)	1.12 (0.93, 1.33)	1.10 (0.93, 1.31)	2.33 (-3.71, 8.37)	3.63 (-2.34, 9.61)	3.24 (-2.56, 9.04)
Sanitation	514	22.2%	0.70 (0.58, 0.85)	0.71 (0.60, 0.85)	0.70 (0.59, 0.83)	-9.59 (-14.62, -4.55)	-9.02 (-13.68, -4.35)	-9.32 (-13.76, -4.87)
Handwashing	498	28.3%	0.89 (0.72, 1.10)	0.87 (0.71, 1.07)	0.87 (0.71, 1.05)	-3.47 (-9.78, 2.84)	-4.00 (-9.84, 1.83)	-4.21 (-9.88, 1.47)
WSH	479	26.1%	0.82 (0.68, 0.99)	0.83 (0.69, 1.00)	0.81 (0.67, 0.99)	-5.71 (-10.89, -0.52)	-5.32 (-10.45, -0.19)	-5.94 (-11.30, -0.58)
Nutrition	447	31.1%	0.98 (0.81, 1.17)	0.96 (0.80, 1.15)	0.97 (0.81, 1.16)	-0.76 (-6.45, 4.93)	-1.31 (-6.95, 4.34)	-1.01 (-6.50, 4.48)
Nutrition + WSH	473	24.9%	0.78 (0.64, 0.96)	0.81 (0.67, 0.98)	0.81 (0.67, 0.97)	-6.85 (-12.16, -1.54)	-6.02 (-11.13, -0.90)	-6.04 (-11.15, -0.93)
E. histolytica								
Control	802	0.0%						
Water	506	0.6%	– [c]	– [c]	– [c]	– [c]	– [c]	– [c]
Sanitation	514	0.6%	– [c]	– [c]	– [c]	– [c]	– [c]	– [c]
Handwashing	498	0.8%	– [c]	– [c]	– [c]	– [c]	– [c]	– [c]
WSH	479	0.0%	– [c]	– [c]	– [c]	– [c]	– [c]	– [c]
Nutrition	447	0.7%	– [c]	– [c]	– [c]	– [c]	– [c]	– [c]
Nutrition + WSH	473	0.4%	– [c]	– [c]	– [c]	– [c]	– [c]	– [c]
Cryptosporidium								
Control	802	1.4%						
Water	506	1.2%	0.86 (0.21, 3.60)	0.86 (0.21, 3.51)	0.87 (0.28, 2.73)	-0.19 (-1.94, 1.57)	-0.20 (-1.94, 1.55)	-0.17 (-1.60, 1.26)
Sanitation	514	1.2%	0.84 (0.31, 2.27)	0.92 (0.36, 2.38)	0.93 (0.36, 2.37)	-0.23 (-1.48, 1.02)	-0.11 (-1.32, 1.11)	-0.09 (-1.23, 1.05)
Handwashing	498	2.0%	1.47 (0.62, 3.46)	1.53 (0.69, 3.40)	1.53 (0.71, 3.26)	0.64 (-0.82, 2.11)	0.71 (-0.68, 2.10)	0.72 (-0.63, 2.07)
WSH	479	1.3%	0.91 (0.26, 3.17)	0.99 (0.29, 3.36)	0.99 (0.34, 2.89)	-0.13 (-1.75, 1.50)	-0.02 (-1.67, 1.63)	-0.02 (-1.42, 1.39)
Nutrition	447	0.9%	0.64 (0.18, 2.29)	0.64 (0.19, 2.21)	0.66 (0.18, 2.44)	-0.50 (-1.88, 0.89)	-0.50 (-1.86, 0.86)	-0.46 (-1.83, 0.92)
Nutrition + WSH	473	1.5%	1.08 (0.41, 2.83)	1.09 (0.43, 2.78)	1.12 (0.44, 2.84)	0.11 (-1.26, 1.48)	0.12 (-1.22, 1.46)	0.16 (-1.21, 1.53)
**Any protozoa**								
Control	802	32.7%						
Water	506	35.6%	1.09 (0.91, 1.29)	1.13 (0.95, 1.34)	1.11 (0.94, 1.32)	2.85 (-3.13, 8.83)	4.03 (-1.93, 9.99)	3.70 (-2.06, 9.46)
Sanitation	514	23.5%	0.72 (0.60, 0.87)	0.73 (0.62, 0.87)	0.72 (0.61, 0.86)	-9.11 (-14.27, -3.96)	-8.60 (-13.34, -3.85)	-8.93 (-13.54, -4.33)
Handwashing	498	29.7%	0.91 (0.74, 1.12)	0.89 (0.73, 1.09)	0.89 (0.73, 1.08)	-2.93 (-9.29, 3.44)	-3.48 (-9.43, 2.47)	-3.59 (-9.41, 2.23)
WSH	479	27.3%	0.84 (0.70, 1.00)	0.85 (0.71, 1.01)	0.83 (0.68, 1.00)	-5.33 (-10.60, -0.07)	-4.94 (-10.20, 0.32)	-5.57 (-11.07, -0.07)
Nutrition	447	32.0%	0.98 (0.82, 1.16)	0.96 (0.81, 1.15)	0.97 (0.81, 1.15)	-0.76 (-6.40, 4.88)	-1.27 (-6.93, 4.38)	-1.05 (-6.55, 4.44)
Nutrition + WSH	473	26.2%	0.80 (0.66, 0.97)	0.82 (0.69, 0.99)	0.83 (0.69, 0.99)	-6.45 (-11.80, -1.11)	-5.68 (-10.87, -0.49)	-5.60 (-10.77, -0.43)
**Multiple protozoa**								
Control	802	0.5%						
Water	506	0.2%	0.38 (0.01, 11.45)	0.40 (0.01, 11.60)	0.40 (0.01, 11.60)	-0.31 (-1.18, 0.56)	-0.30 (-1.18, 0.57)	-0.30 (-1.18, 0.57)
Sanitation	514	0.4%	0.78 (0.14, 4.45)	0.86 (0.17, 4.39)	0.89 (0.18, 4.33)	-0.11 (-0.88, 0.66)	-0.07 (-0.79, 0.66)	-0.05 (-0.76, 0.66)
Handwashing	498	1.4%	2.82 (0.65, 12.33)	3.02 (0.77, 11.83)	3.02 (0.77, 11.83)	0.91 (-0.32, 2.14)	0.97 (-0.19, 2.14)	0.97 (-0.19, 2.14)
WSH	479	0.0%	– [c]	– [c]	– [c]	-0.50 (-1.21, 0.21)	-0.50 (-1.19, 0.20)	-0.50 (-1.21, 0.21)
Nutrition	447	0.7%	1.33 (0.24, 7.28)	1.38 (0.30, 6.42)	1.42 (0.31, 6.62)	0.16 (-0.80, 1.13)	0.19 (-0.68, 1.07)	0.21 (-0.67, 1.08)
Nutrition + WSH	473	0.6%	1.25 (0.23, 6.91)	1.46 (0.31, 6.79)	1.46 (0.31, 6.79)	0.13 (-0.83, 1.08)	0.22 (-0.66, 1.10)	0.22 (-0.66, 1.10)

[a] See pre-specified analysis plan for adjustment covariates.
[b] Inverse probability of censoring weighting.
[c] Could not calculate due to sparse data.

Table S9: Protozoa prevalence among index children approximately 2.5 years after intervention initiation, combined vs. individual WSH

			Prevalence ratio			Prevalence difference		
Arm	N	Prev	Unadjusted	Adjusted [a]	IPCW [b]	Unadjusted	Adjusted [a]	IPCW [b]
Giardia								
WSH	479	26.1%						
Water	506	34.2%	0.76 (0.61, 0.96)	0.74 (0.59, 0.92)	0.74 (0.60, 0.91)	-8.10 (-14.80, -1.40)	-9.03 (-15.64, -2.42)	-9.13 (-15.43, -2.82)
Sanitation	514	22.2%	1.17 (0.94, 1.46)	1.16 (0.94, 1.44)	1.15 (0.93, 1.43)	3.84 (-1.48, 9.16)	3.61 (-1.64, 8.86)	3.42 (-1.82, 8.66)
Handwashing	498	28.3%	0.92 (0.73, 1.16)	0.94 (0.74, 1.19)	0.93 (0.73, 1.19)	-2.24 (-8.62, 4.14)	-1.61 (-7.98, 4.76)	-1.87 (-8.40, 4.65)
E. histolytica								
WSH	479	0.0%						
Water	506	0.6%	– [c]	– [c]	– [c]	-0.61 (-1.35, 0.12)	-0.60 (-1.34, 0.15)	-0.59 (-1.26, 0.08)
Sanitation	514	0.6%	– [c]	– [c]	– [c]	-0.59 (-1.25, 0.06)	-0.58 (-1.22, 0.05)	-0.60 (-1.23, 0.02)
Handwashing	498	0.8%	– [c]	– [c]	– [c]	-0.80 (-1.80, 0.20)	-0.80 (-1.84, 0.24)	-0.81 (-1.78, 0.16)
Cryptosporidium								
WSH	479	1.3%						
Water	506	1.2%	1.06 (0.34, 3.29)	1.08 (0.37, 3.21)	1.00 (0.40, 2.52)	0.07 (-1.33, 1.46)	0.10 (-1.27, 1.47)	0.00 (-1.14, 1.14)
Sanitation	514	1.2%	1.06 (0.35, 3.25)	1.05 (0.34, 3.23)	1.04 (0.36, 3.02)	0.07 (-1.28, 1.42)	0.05 (-1.34, 1.45)	0.05 (-1.26, 1.35)
Handwashing	498	2.0%	0.64 (0.21, 2.00)	0.63 (0.20, 2.05)	0.63 (0.22, 1.85)	-0.71 (-2.48, 1.06)	-0.74 (-2.61, 1.13)	-0.76 (-2.53, 1.01)
**Any protozoa**								
WSH	479	27.3%						
Water	506	35.6%	0.77 (0.62, 0.96)	0.75 (0.60, 0.93)	0.74 (0.60, 0.91)	-8.22 (-14.99, -1.46)	-9.15 (-15.81, -2.48)	-9.36 (-15.76, -2.96)
Sanitation	514	23.5%	1.16 (0.94, 1.43)	1.14 (0.93, 1.40)	1.14 (0.93, 1.40)	3.69 (-1.69, 9.07)	3.40 (-1.87, 8.66)	3.32 (-1.92, 8.56)
Handwashing	498	29.7%	0.92 (0.73, 1.15)	0.94 (0.75, 1.18)	0.93 (0.74, 1.17)	-2.36 (-8.80, 4.07)	-1.76 (-8.30, 4.78)	-2.08 (-8.69, 4.53)
**Multiple protozoa**								
WSH	479	0.0%						
Water	506	0.2%	– [c]	– [c]	– [c]	-0.20 (-0.59, 0.19)	-0.19 (-0.58, 0.21)	-0.19 (-0.58, 0.21)
Sanitation	514	0.4%	– [c]	– [c]	– [c]	-0.38 (-0.91, 0.15)	-0.39 (-0.94, 0.15)	-0.39 (-0.94, 0.16)
Handwashing	498	1.4%	– [c]	– [c]	– [c]	-1.39 (-2.40, -0.37)	-1.42 (-2.50, -0.35)	-1.42 (-2.50, -0.35)

[a] See pre-specified analysis plan for adjustment covariates.
[b] Inverse probability of censoring weighting.
[c] Could not calculate due to sparse data.

Table S10: Protozoa prevalence among index children approximately 2.5 years after intervention initiation, combined nutrition plus WSH vs. WSH and nutrition

			Prevalence ratio			Prevalence difference		
Arm	N	Prev	Unadjusted	Adjusted [a]	IPCW [b]	Unadjusted	Adjusted [a]	IPCW [b]
Giardia								
Nutrition + WSH	473	24.9%						
WSH	479	26.1%	0.96 (0.75, 1.23)	0.96 (0.75, 1.23)	0.97 (0.77, 1.23)	-1.06 (-7.44, 5.33)	-0.94 (-7.18, 5.30)	-0.74 (-6.77, 5.29)
Nutrition	447	31.1%	0.81 (0.66, 0.99)	0.82 (0.67, 0.99)	0.81 (0.67, 0.98)	-6.01 (-11.62, -0.40)	-5.68 (-11.14, -0.23)	-5.86 (-10.95, -0.77)
E. histolytica								
Nutrition + WSH	473	0.4%						
WSH	479	0.0%	– [c]	– [c]	– [c]	0.43 (-0.09, 0.95)	0.42 (-0.07, 0.91)	0.46 (-0.10, 1.03)
Nutrition	447	0.7%	0.63 (0.10, 3.83)	0.64 (0.11, 3.83)	0.63 (0.11, 3.50)	-0.25 (-1.31, 0.80)	-0.24 (-1.29, 0.80)	-0.25 (-1.19, 0.68)
Cryotosporidium								
Nutrition + WSH	473	1.5%						
WSH	479	1.3%	1.18 (0.34, 4.11)	1.13 (0.33, 3.83)	1.17 (0.38, 3.61)	0.22 (-1.46, 1.91)	0.16 (-1.50, 1.83)	0.22 (-1.35, 1.79)
Nutrition	447	0.9%	1.67 (0.43, 6.39)	1.59 (0.43, 5.90)	1.58 (0.41, 6.14)	0.60 (-0.87, 2.06)	0.54 (-0.91, 1.99)	0.53 (-1.00, 2.07)
**Any protozoa**								
Nutrition + WSH	473	26.2%						
WSH	479	27.3%	0.96 (0.76, 1.21)	0.97 (0.77, 1.21)	0.98 (0.78, 1.22)	-1.04 (-7.23, 5.16)	-0.92 (-6.96, 5.12)	-0.62 (-6.60, 5.36)
Nutrition	447	32.0%	0.82 (0.68, 1.00)	0.83 (0.69, 1.00)	0.83 (0.70, 0.98)	-5.61 (-11.11, -0.12)	-5.35 (-10.68, -0.02)	-5.50 (-10.46, -0.54)
**Multiple protozoa**								
Nutrition + WSH	473	0.6%						
WSH	479	0.0%	– [c]	– [c]	– [c]	0.64 (-0.07, 1.36)	0.63 (-0.05, 1.31)	0.73 (0.04, 1.41)
Nutrition	447	0.7%	0.92 (0.19, 4.48)	0.95 (0.20, 4.55)	0.91 (0.19, 4.31)	-0.05 (-1.09, 0.98)	-0.03 (-1.07, 1.00)	-0.06 (-1.09, 0.96)

[a] See pre-specified analysis plan for adjustment covariates.
[b] Inverse probability of censoring weighting.
[c] Could not calculate due to sparse data.

Table S11: Ct reduction [a] among index children approximately 2.5 years after intervention initiation, all interventions vs. control

Arm	N	Mean	Unadjusted	Adjusted [b]	IPCW [c]
Giardia					
Control	802	37.0			
Water	506	36.9	-0.00 (-0.02, 0.01)	-0.01 (-0.02, 0.01)	-0.01 (-0.02, 0.01)
Sanitation	514	38.0	0.03 (0.01, 0.04)	0.02 (0.01, 0.04)	0.02 (0.01, 0.04)
Handwashing	498	37.2	0.01 (-0.01, 0.02)	0.01 (-0.01, 0.03)	0.01 (-0.01, 0.02)
WSH	479	37.6	0.01 (-0.00, 0.03)	0.01 (-0.00, 0.03)	0.02 (0.00, 0.03)
Nutrition	447	37.2	0.01 (-0.01, 0.02)	0.01 (-0.01, 0.02)	0.01 (-0.01, 0.02)
Nutrition + WSH	473	37.8	0.02 (0.01, 0.04)	0.02 (0.00, 0.04)	0.02 (0.00, 0.04)
E. histolytica [d]					
Control	802	40.0			
Water	506	40.0	–	–	–
Sanitation	514	40.0	–	–	–
Handwashing	498	40.0	–	–	–
WSH	479	40.0	–	–	–
Nutrition	447	40.0	–	–	–
Nutrition + WSH	473	40.0	–	–	–
Cryptosporidium [d]					
Control	802	39.9			
Water	506	39.9	–	–	–
Sanitation	514	39.9	–	–	–
Handwashing	498	39.9	–	–	–
WSH	479	39.9	–	–	–
Nutrition	447	40.0	–	–	–
Nutrition + WSH	473	39.9	–	–	–

[a] Ct reduction defined as Ct ratio (CtR) - 1, where CtR is the ratio of Ct values between arms.
[b] See pre-specified analysis plan for adjustment covariates.
[c] Inverse probability of censoring weighting.
[d] Could not calculate due to sparse data.

Table S12: Ct reduction [a] among index children approximately 2.5 years after intervention initiation, combined vs. individual WSH

Arm	N	Mean	Unadjusted	Adjusted [b]	IPCW [c]
Giardia					
WSH	479	37.6			
Water	506	36.9	0.02 (-0.00, 0.04)	0.02 (0.00, 0.04)	0.02 (0.00, 0.04)
Sanitation	514	38.0	-0.01 (-0.03, 0.01)	-0.01 (-0.03, 0.01)	-0.01 (-0.03, 0.01)
Handwashing	498	37.2	0.01 (-0.01, 0.03)	0.01 (-0.01, 0.03)	0.01 (-0.01, 0.03)
E. histolytica [d]					
WSH	479	40.0			
Water	506	40.0	–	–	–
Sanitation	514	40.0	–	–	–
Handwashing	498	40.0	–	–	–
Cryptosporidium [d]					
WSH	479	39.9			
Water	506	39.9	–	–	–
Sanitation	514	39.9	–	–	–
Handwashing	498	39.9	–	–	–

[a] Ct reduction defined as Ct ratio (CtR) - 1, where CtR is the ratio of Ct values between arms.
[b] See pre-specified analysis plan for adjustment covariates.
[c] Inverse probability of censoring weighting.
[d] Could not calculate due to sparse data.

Table S13: Ct reduction [a] among index children approximately 2.5 years after intervention initiation, combined nutrition plus WSH vs. WSH and nutrition

Arm	N	Mean	Unadjusted	Adjusted [b]	IPCW [c]
Giardia					
Nutrition + WSH	473	37.8			
WSH	479	37.6	0.01 (-0.01, 0.03)	0.01 (-0.01, 0.02)	0.00 (-0.01, 0.02)
Nutrition	447	37.2	0.02 (-0.00, 0.03)	0.02 (-0.00, 0.03)	0.02 (-0.00, 0.03)
E. histolytica [d]					
Nutrition + WSH	473	40.0			
WSH	479	40.0	–	–	–
Nutrition	447	40.0	–	–	–
Cryptosporidium [d]					
Nutrition + WSH	473	39.9			
WSH	479	39.9	–	–	–
Nutrition	447	40.0	–	–	–

[a] Ct reduction defined as Ct ratio (CtR) - 1, where CtR is the ratio of Ct values between arms.
[b] See pre-specified analysis plan for adjustment covariates.
[c] Inverse probability of censoring weighting.
[c] Could not calculate due to sparse data.

Table S14: Protozoa prevalence among all observations approximately 2.5 years after intervention initiation, all interventions vs. control

			Prevalence ratio			Prevalence difference		
Arm	N	Prev	Unadjusted	Adjusted [a]	IPCW [b]	Unadjusted	Adjusted [a]	IPCW [b]
Giardia								
Control	1468	35.4%						
Water	926	37.4%	1.05 (0.91, 1.22)	1.08 (0.94, 1.25)	1.08 (0.93, 1.24)	1.93 (-3.43, 7.30)	2.91 (-2.41, 8.23)	2.70 (-2.56, 7.95)
Sanitation	934	27.3%	0.77 (0.67, 0.89)	0.78 (0.69, 0.89)	0.78 (0.68, 0.88)	-8.05 (-12.55, -3.55)	-7.68 (-11.76, -3.60)	-7.89 (-11.87, -3.92)
Handwashing	939	28.9%	0.82 (0.69, 0.96)	0.81 (0.70, 0.95)	0.80 (0.69, 0.93)	-6.49 (-11.55, -1.43)	-6.52 (-11.33, -1.71)	-6.89 (-11.48, -2.29)
WSH	908	30.6%	0.87 (0.76, 0.99)	0.88 (0.77, 0.99)	0.87 (0.76, 0.99)	-4.74 (-8.93, -0.54)	-4.38 (-8.51, -0.24)	-4.73 (-9.08, -0.39)
Nutrition	824	33.1%	0.94 (0.81, 1.08)	0.92 (0.81, 1.06)	0.92 (0.81, 1.06)	-2.21 (-6.98, 2.56)	-2.66 (-7.30, 1.97)	-2.66 (-7.24, 1.93)
Nutrition + WSH	900	28.1%	0.80 (0.69, 0.92)	0.82 (0.72, 0.94)	0.81 (0.71, 0.92)	-7.24 (-11.61, -2.88)	-6.29 (-10.42, -2.15)	-6.66 (-10.62, -2.70)
E. histolytica								
Control	1468	0.5%						
Water	926	0.3%	0.59 (0.12, 2.83)	0.60 (0.12, 2.90)	0.55 (0.15, 2.08)	-0.23 (-0.84, 0.38)	-0.22 (-0.83, 0.39)	-0.26 (-0.84, 0.33)
Sanitation	934	0.6%	1.17 (0.40, 3.45)	1.16 (0.40, 3.42)	1.07 (0.35, 3.28)	0.09 (-0.55, 0.73)	0.09 (-0.55, 0.73)	0.04 (-0.67, 0.76)
Handwashing	939	1.3%	2.36 (0.91, 6.14)	2.36 (0.93, 6.02)	2.33 (0.87, 6.26)	0.74 (-0.13, 1.60)	0.74 (-0.11, 1.59)	0.75 (-0.12, 1.62)
WSH	908	0.0%	– [c]	– [c]	– [c]	-0.54 (-1.00, -0.09)	-0.50 (-0.91, -0.09)	-0.56 (-1.03, -0.09)
Nutrition	824	1.3%	2.44 (1.09, 5.46)	2.48 (1.19, 5.19)	2.29 (1.06, 4.92)	0.79 (-0.05, 1.62)	0.82 (0.02, 1.62)	0.73 (-0.06, 1.52)
Nutrition + WSH	900	0.2%	0.41 (0.06, 2.64)	0.47 (0.08, 2.77)	0.39 (0.07, 2.31)	-0.32 (-0.96, 0.32)	-0.27 (-0.89, 0.35)	-0.33 (-0.94, 0.27)
Cryptosporidium								
Control	1468	1.2%						
Water	926	0.8%	0.65 (0.18, 2.41)	0.63 (0.18, 2.24)	0.65 (0.24, 1.72)	-0.40 (-1.52, 0.71)	-0.43 (-1.50, 0.63)	-0.40 (-1.25, 0.45)
Sanitation	934	1.0%	0.83 (0.36, 1.94)	0.88 (0.40, 1.93)	0.92 (0.42, 2.01)	-0.19 (-1.08, 0.69)	-0.14 (-0.96, 0.69)	-0.09 (-0.93, 0.75)
Handwashing	939	1.2%	1.01 (0.44, 2.30)	1.06 (0.48, 2.31)	1.07 (0.53, 2.18)	0.01 (-0.95, 0.97)	0.07 (-0.85, 0.98)	0.08 (-0.76, 0.92)
WSH	908	0.8%	0.67 (0.21, 2.15)	0.71 (0.24, 2.06)	0.72 (0.28, 1.86)	-0.39 (-1.47, 0.70)	-0.34 (-1.36, 0.68)	-0.32 (-1.22, 0.58)
Nutrition	824	0.6%	0.52 (0.15, 1.78)	0.51 (0.17, 1.58)	0.55 (0.17, 1.77)	-0.55 (-1.55, 0.45)	-0.57 (-1.49, 0.36)	-0.53 (-1.46, 0.41)
Nutrition + WSH	900	1.0%	0.86 (0.37, 2.03)	0.99 (0.45, 2.14)	0.95 (0.43, 2.10)	-0.16 (-1.07, 0.75)	-0.02 (-0.87, 0.84)	-0.05 (-0.92, 0.82)
**Any protozoa**								
Control	1468	36.3%						
Water	926	38.2%	1.05 (0.91, 1.22)	1.08 (0.93, 1.24)	1.07 (0.93, 1.23)	1.85 (-3.60, 7.30)	2.75 (-2.68, 8.19)	2.54 (-2.80, 7.87)
Sanitation	934	28.4%	0.78 (0.67, 0.91)	0.79 (0.69, 0.90)	0.78 (0.69, 0.89)	-7.93 (-12.63, -3.23)	-7.59 (-11.84, -3.33)	-7.85 (-12.05, -3.65)
Handwashing	939	30.2%	0.83 (0.71, 0.98)	0.83 (0.71, 0.97)	0.82 (0.71, 0.95)	-6.06 (-11.19, -0.93)	-6.14 (-11.06, -1.21)	-6.43 (-11.15, -1.71)
WSH	908	31.4%	0.86 (0.76, 0.99)	0.87 (0.77, 0.99)	0.86 (0.75, 0.99)	-4.92 (-9.30, -0.54)	-4.62 (-8.95, -0.30)	-4.99 (-9.47, -0.51)
Nutrition	824	34.3%	0.95 (0.82, 1.09)	0.94 (0.82, 1.07)	0.94 (0.82, 1.07)	-1.95 (-6.79, 2.89)	-2.32 (-7.01, 2.37)	-2.31 (-6.94, 2.33)
Nutrition + WSH	900	28.8%	0.79 (0.69, 0.91)	0.82 (0.72, 0.93)	0.81 (0.71, 0.91)	-7.53 (-11.91, -3.15)	-6.52 (-10.65, -2.39)	-6.93 (-10.90, -2.97)
**Multiple protozoa**								
Control	1468	0.7%						
Water	926	0.1%	0.14 (0.00, 4.74)	0.15 (0.00, 4.78)	0.15 (0.00, 4.79)	-0.65 (-1.27, -0.03)	-0.64 (-1.26, -0.01)	-0.63 (-1.25, -0.01)
Sanitation	934	0.5%	0.72 (0.30, 1.71)	0.77 (0.35, 1.68)	0.78 (0.36, 1.71)	-0.21 (-0.76, 0.34)	-0.17 (-0.67, 0.33)	-0.16 (-0.66, 0.34)
Handwashing	939	1.1%	1.42 (0.56, 3.58)	1.53 (0.65, 3.63)	1.52 (0.64, 3.60)	0.31 (-0.51, 1.14)	0.39 (-0.40, 1.18)	0.38 (-0.41, 1.16)
WSH	908	0.0%	– [c]	– [c]	– [c]	-0.75 (-1.30, -0.20)	-0.73 (-1.24, -0.21)	-0.73 (-1.24, -0.21)
Nutrition	824	0.7%	0.96 (0.33, 2.80)	0.99 (0.39, 2.53)	1.04 (0.39, 2.73)	-0.03 (-0.81, 0.76)	-0.01 (-0.70, 0.69)	0.03 (-0.70, 0.75)
Nutrition + WSH	900	0.6%	0.74 (0.26, 2.15)	0.87 (0.33, 2.31)	0.82 (0.31, 2.15)	-0.19 (-0.87, 0.48)	-0.09 (-0.74, 0.56)	-0.13 (-0.76, 0.50)

[a] See pre-specified analysis plan for adjustment covariates.

[b] Inverse probability of censoring weighting.

[c] Could not calculate due to sparse data.

Table S15: Protozoa prevalence among all observations approximately 2.5 years after intervention initiation, combined vs. individual WSH

			Prevalence ratio			Prevalence difference		
Arm	N	Prev	Unadjusted	Adjusted [a]	IPCW [b]	Unadjusted	Adjusted [a]	IPCW [b]
Giardia								
WSH	908	30.6%						
Water	926	37.4%	0.82 (0.71, 0.95)	0.80 (0.69, 0.93)	0.80 (0.69, 0.92)	-6.73 (-11.78, -1.69)	-7.50 (-12.45, -2.55)	-7.62 (-12.50, -2.75)
Sanitation	934	27.3%	1.12 (0.96, 1.32)	1.12 (0.96, 1.31)	1.11 (0.95, 1.30)	3.32 (-1.32, 7.96)	3.39 (-1.12, 7.90)	3.11 (-1.41, 7.62)
Handwashing	939	28.9%	1.06 (0.91, 1.24)	1.08 (0.92, 1.26)	1.08 (0.92, 1.26)	1.76 (-2.90, 6.42)	2.20 (-2.46, 6.85)	2.20 (-2.53, 6.92)
E. histolytica								
WSH	908	0.0%						
Water	926	0.3%	– [c]	– [c]	– [c]	-0.33 (-0.77, 0.11)	-0.32 (-0.76, 0.11)	-0.32 (-0.70, 0.07)
Sanitation	934	0.6%	– [c]	– [c]	– [c]	-0.64 (-1.21, -0.08)	-0.68 (-1.25, -0.11)	-0.66 (-1.22, -0.10)
Handwashing	939	1.3%	– [c]	– [c]	– [c]	-1.28 (-2.09, -0.47)	-1.33 (-2.18, -0.49)	-1.34 (-2.14, -0.55)
Cryptosporidium								
WSH	908	0.8%						
Water	926	0.8%	1.02 (0.41, 2.51)	1.04 (0.43, 2.50)	1.01 (0.46, 2.24)	0.01 (-0.68, 0.71)	0.03 (-0.65, 0.71)	0.01 (-0.60, 0.61)
Sanitation	934	1.0%	0.76 (0.26, 2.20)	0.78 (0.26, 2.27)	0.80 (0.32, 2.03)	-0.23 (-1.12, 0.66)	-0.22 (-1.13, 0.69)	-0.19 (-0.99, 0.61)
Handwashing	939	1.2%	0.66 (0.22, 1.98)	0.67 (0.22, 2.06)	0.67 (0.25, 1.80)	-0.40 (-1.45, 0.64)	-0.38 (-1.46, 0.69)	-0.39 (-1.35, 0.58)
**Any protozoa**								
WSH	908	31.4%						
Water	926	38.2%	0.82 (0.71, 0.95)	0.80 (0.70, 0.93)	0.80 (0.69, 0.92)	-6.83 (-11.97, -1.69)	-7.60 (-12.65, -2.54)	-7.73 (-12.67, -2.78)
Sanitation	934	28.4%	1.11 (0.94, 1.30)	1.11 (0.95, 1.29)	1.10 (0.94, 1.28)	2.99 (-1.79, 7.77)	3.03 (-1.60, 7.66)	2.80 (-1.80, 7.40)
Handwashing	939	30.2%	1.04 (0.89, 1.21)	1.05 (0.90, 1.23)	1.04 (0.89, 1.22)	1.14 (-3.61, 5.90)	1.53 (-3.26, 6.31)	1.35 (-3.50, 6.20)
**Multiple protozoa**								
WSH	908	0.0%						
Water	926	0.1%	– [c]	– [c]	– [c]	-0.11 (-0.33, 0.11)	-0.10 (-0.31, 0.12)	-0.10 (-0.31, 0.12)
Sanitation	934	0.5%	– [c]	– [c]	– [c]	-0.54 (-0.99, -0.08)	-0.55 (-1.01, -0.09)	-0.55 (-1.01, -0.10)
Handwashing	939	1.1%	– [c]	– [c]	– [c]	-1.07 (-1.69, -0.44)	-1.08 (-1.73, -0.43)	-1.08 (-1.73, -0.43)

[a] See pre-specified analysis plan for adjustment covariates.
[b] Inverse probability of censoring weighting.
[c] Could not calculate due to sparse data.

Table S16: Protozoa prevalence among all observations approximately 2.5 years after intervention initiation, combined nutrition plus WSH vs. WSH and nutrition

			Prevalence ratio			Prevalence difference		
Arm	N	Prev	Unadjusted	Adjusted [a]	IPCW [b]	Unadjusted	Adjusted [a]	IPCW [b]
Giardia								
Nutrition + WSH	900	28.1%						
WSH	908	30.6%	0.92 (0.78, 1.09)	0.93 (0.79, 1.09)	0.93 (0.80, 1.09)	-2.45 (-7.34, 2.45)	-2.27 (-7.07, 2.54)	-2.07 (-6.70, 2.56)
Nutrition	824	33.1%	0.85 (0.73, 0.99)	0.87 (0.75, 1.00)	0.87 (0.75, 1.00)	-5.02 (-9.64, -0.39)	-4.22 (-8.57, 0.13)	-4.35 (-8.64, -0.05)
E. histolytica								
Nutrition + WSH	900	0.2%						
WSH	908	0.0%	– [c]	– [c]	– [c]	0.23 (-0.08, 0.54)	0.24 (-0.04, 0.52)	0.25 (-0.06, 0.56)
Nutrition	824	1.3%	0.17 (0.04, 0.77)	0.18 (0.04, 0.82)	0.17 (0.03, 0.87)	-1.11 (-2.25, 0.03)	-1.02 (-2.11, 0.07)	-1.10 (-2.09, -0.11)
Cryotosporidium								
Nutrition + WSH	900	1.0%						
WSH	908	0.8%	1.29 (0.38, 4.36)	1.27 (0.39, 4.17)	1.30 (0.44, 3.86)	0.23 (-0.84, 1.29)	0.21 (-0.83, 1.25)	0.23 (-0.75, 1.21)
Nutrition	824	0.6%	1.65 (0.45, 6.07)	1.71 (0.46, 6.36)	1.62 (0.41, 6.46)	0.39 (-0.56, 1.35)	0.43 (-0.54, 1.39)	0.38 (-0.66, 1.42)
**Any protozoa**								
Nutrition + WSH	900	28.8%						
WSH	908	31.4%	0.92 (0.78, 1.08)	0.92 (0.79, 1.08)	0.93 (0.80, 1.08)	-2.54 (-7.30, 2.21)	-2.42 (-7.12, 2.29)	-2.13 (-6.69, 2.44)
Nutrition	824	34.3%	0.84 (0.73, 0.97)	0.86 (0.75, 0.99)	0.86 (0.75, 0.98)	-5.57 (-10.05, -1.08)	-4.70 (-8.92, -0.48)	-4.91 (-9.09, -0.73)
**Multiple protozoa**								
Nutrition + WSH	900	0.6%						
WSH	908	0.0%	– [c]	– [c]	– [c]	0.55 (0.05, 1.05)	0.59 (0.09, 1.08)	0.57 (0.07, 1.06)
Nutrition	824	0.7%	0.77 (0.27, 2.15)	0.84 (0.30, 2.34)	0.74 (0.26, 2.08)	-0.17 (-0.83, 0.49)	-0.11 (-0.76, 0.54)	-0.19 (-0.86, 0.47)

[a] See pre-specified analysis plan for adjustment covariates.
[b] Inverse probability of censoring weighting.
[c] Could not calculate due to sparse data.

Table S17: Ct reduction [a] among all observations approximately 2.5 years after intervention initiation, all interventions vs. control

Arm	N	Mean	Unadjusted	Adjusted [b]	IPCW [c]
Giardia					
Control	1468	36.7			
Water	926	36.3	-0.01 (-0.02, 0.01)	-0.01 (-0.03, 0.01)	-0.01 (-0.03, 0.01)
Sanitation	934	37.5	0.02 (0.01, 0.04)	0.02 (0.01, 0.03)	0.02 (0.01, 0.03)
Handwashing	939	37.3	0.02 (0.00, 0.03)	0.02 (0.00, 0.03)	0.02 (0.00, 0.03)
WSH	908	37.2	0.02 (0.00, 0.03)	0.02 (0.00, 0.03)	0.02 (0.00, 0.03)
Nutrition	824	37.0	0.01 (-0.00, 0.02)	0.01 (-0.00, 0.02)	0.01 (-0.00, 0.02)
Nutrition + WSH	900	37.4	0.02 (0.01, 0.04)	0.02 (0.01, 0.03)	0.02 (0.01, 0.03)
E. histolytica [d]					
Control	1468	40.0			
Water	926	40.0	–	–	–
Sanitation	934	40.0	–	–	–
Handwashing	939	39.9	–	–	–
WSH	908	40.0	–	–	–
Nutrition	824	39.9	–	–	–
Nutrition + WSH	900	40.0	–	–	–
Cryptosporidium [d]					
Control	1468	39.9			
Water	926	39.9	–	–	–
Sanitation	934	39.9	–	–	–
Handwashing	939	39.9	–	–	–
WSH	908	39.9	–	–	–
Nutrition	824	40.0	–	–	–
Nutrition + WSH	900	39.9	–	–	–

[a] Ct reduction defined as Ct ratio (CtR) - 1, where CtR is the ratio of Ct values between arms.
[b] See pre-specified analysis plan for adjustment covariates.
[c] Inverse probability of censoring weighting.
[d] Could not calculate due to sparse data.

Table S18: Ct reduction [a] among all observations approximately 2.5 years after intervention initiation, combined vs. individual WSH

Arm	N	Mean	Unadjusted	Adjusted [b]	IPCW [c]
Giardia					
WSH	908	37.2			
Water	926	36.3	0.02 (0.01, 0.04)	0.03 (0.01, 0.04)	0.03 (0.01, 0.04)
Sanitation	934	37.5	-0.01 (-0.02, 0.01)	-0.01 (-0.02, 0.01)	-0.01 (-0.02, 0.01)
Handwashing	939	37.3	-0.00 (-0.01, 0.01)	-0.00 (-0.02, 0.01)	-0.00 (-0.01, 0.01)
E. histolytica [d]					
WSH	908	40.0			
Water	926	40.0	–	–	–
Sanitation	934	40.0	–	–	–
Handwashing	939	39.9	–	–	–
Cryptosporidium [d]					
WSH	908	39.9			
Water	926	39.9	–	–	–
Sanitation	934	39.9	–	–	–
Handwashing	939	39.9	–	–	–

[a] Ct reduction defined as Ct ratio (CtR) - 1, where CtR is the ratio of Ct values between arms.
[b] See pre-specified analysis plan for adjustment covariates.
[c] Inverse probability of censoring weighting.
[d] Could not calculate due to sparse data.

Table S19: Ct reduction [a] among all observations approximately 2.5 years after intervention initiation, combined nutrition plus WSH vs. WSH and nutrition

Arm	N	Mean	Unadjusted	Adjusted [b]	IPCW [c]
Giardia					
Nutrition + WSH	900	37.4			
WSH	908	37.2	0.01 (-0.01, 0.02)	0.00 (-0.01, 0.02)	0.00 (-0.01, 0.02)
Nutrition	824	37.0	0.01 (-0.00, 0.03)	0.01 (-0.00, 0.02)	0.01 (-0.00, 0.02)
E. histolytica [d]					
Nutrition + WSH	900	40.0			
WSH	908	40.0	–	–	–
Nutrition	824	39.9	–	–	–
Cryptosporidium [d]					
Nutrition + WSH	900	39.9			
WSH	908	39.9	–	–	–
Nutrition	824	40.0	–	–	–

[a] Ct reduction defined as Ct ratio (CtR) - 1, where CtR is the ratio of Ct values between arms.
[b] See pre-specified analysis plan for adjustment covariates.
[c] Inverse probability of censoring weighting.
[d] Could not calculate due to sparse data.

Table S20: Ct reduction[a] among index households approximately 2.5 years after intervention initiation, analysis restricted to specimens with positive protozoan infections

	Giardia			*E. histolytica*			*Cryptosporidium*		
Arm	N	Mean	Unadjusted	N	Mean	Unadjusted	N	Mean	Unadjusted
**All interventions vs. control**									
Control	451	30.5		4	29.0		16	34.4	
Water	298	30.2	-0.01 (-0.04, 0.02)	3	33.4	_[b]	6	31.9	0.01 (-0.05, 0.07)
Sanitation	218	30.7	0.01 (-0.03, 0.04)	4	34.3	_[b]	9	34.2	0.00 (-0.09, 0.08)
Handwashing	227	30.5	0.00 (-0.03, 0.03)	9	34.1	_[b]	11	33.7	-0.02 (-0.12, 0.09)
WSH	231	30.9	0.02 (-0.02, 0.05)	0	_[b]	_[b]	7	31.6	-0.08 (-0.21, 0.05)
Nutrition	231	30.9	0.01 (-0.01, 0.04)	9	32.9	_[b]	5	35.3	0.02 (-0.05, 0.10)
Nutrition + WSH	200	31.0	0.02 (-0.02, 0.05)	2	35.0	_[b]	9	34.6	0.01 (-0.05, 0.06)
**Combined vs. individual WSH**									
WSH	231	30.9		0	_[b]		7	31.6	
Water	298	30.2	0.02 (-0.01, 0.05)	3	33.4	_[b]	6	31.9	0.01 (-0.13, 0.14)
Sanitation	218	30.7	0.01 (-0.03, 0.04)	4	34.3	_[b]	9	34.2	-0.09 (-0.22, 0.04)
Handwashing	227	30.5	0.02 (-0.01, 0.05)	9	34.1	_[b]	11	33.7	-0.06 (-0.20, 0.07)
**Combined nutrition plus WSH vs. WSH and nutrition**									
Nutrition + WSH	200	31.0		2	35.0	_[b]	9	34.6	
WSH	231	30.9	0.00 (-0.03, 0.03)	7	31.6	_[b]	7	31.6	0.12 (-0.05, 0.28)
Nutrition	231	30.9	0.00 (-0.04, 0.04)	5	35.3	_[b]	5	35.3	-0.02 (-0.09, 0.04)

[a] Ct reduction defined as Ct ratio (CtR) - 1, where CtR is the ratio of Ct values between arms

[b] Could not calculate due to sparse data
