# Supplementary material for: Effects of Water, Sanitation, Handwashing, and Nutritional Interventions on Child Enteric Protozoan Infections in Rural Bangladesh: A Cluster-Randomized Controlled Trial
Source: Clin Infect Dis. 2018 Apr 13;67(10):1515–22. doi: 10.1093/cid/ciy320 (PMC6206106; doi:10.1093/cid/ciy320)
Supplement: Supplementary Material [file ciy320_suppl_supplementary_material.docx]

**Supplementary Material**

**CONSORT checklist**

| **Item** | **Description** | **Reported in Section** |
| --- | --- | --- |
| **Title and Abstract** | | |
| 1a | Identification as a randomized trial in the title; Identification as a cluster randomized trial in the title | Abstract |
| 1b | Structured summary of trial design, methods, results, and conclusions | Abstract |
| **Introduction** | | |
| Background and Objectives | | |
| 2a | Scientific background and explanation of rationale; Rationale for using a cluster design | Introduction |
| 2b | Specific objectives or hypotheses; Whether objectives pertain to the cluster level, the individual participant level, or both | Introduction |
| **Methods** | | |
| Trial Design | | |
| 3a | Description of trial design (such as parallel, factorial) including allocation ratio; Definition of cluster and description of how the design features apply to the clusters | Methods; Supplementary Material (Randomization) |
| 3b | Important changes to methods after trial commencement (such as eligibility criteria), with reasons | N/A |
| Participants | | |
| 4a | Eligibility criteria for participants; Eligibility criteria for clusters | Methods (Participants); Supplementary Materials (Randomization) |
| 4b | Settings and locations where the data were collected | Methods (Participants; Procedures) |
| Interventions | | |
| 5 | The interventions for each group with sufficient details to allow replication, including how and when they were actually administered; Whether interventions pertain to the cluster level, the individual participant level, or both | Methods (Procedures) |
| Outcomes | | |
| 6a | Completely defined pre-specified primary and secondary outcome measures, including how and when they were assessed; Whether outcome measures pertain to the cluster level, the individual participant level, or both | Methods (Registration; Procedures; Outcomes) |
| 6b | Any changes to trial outcomes after the trial commenced, with reasons | N/A |
| Sample Size | | |
| 7a | How sample size was determined; Method of calculation, number of cluster(s) (and whether equal or unequal cluster sizes are assumed), cluster size, a coefficient of intracluster correlation (ICC or k), and an indication of its uncertainty | Methods (Statistical analysis); Supplementary Material |
| 7b | When applicable, explanation of any interim analyses and stopping guidelines | N/A |
| **Randomization** | | |
| Sequence Generation | | |
| 8a | Method used to generate the random allocation sequence | Supplementary Material (Randomization) |
| 8b | Type of randomization; details of any restriction (such as blocking and block size); Details of stratification or matching if used | Supplementary Material (Randomization) |
| Allocation Concealment Mechanism | | |
| 9 | Mechanism used to implement the random allocation sequence (such as sequentially numbered containers), describing any steps taken to conceal the sequence until interventions were assigned; Specification that allocation was based on clusters rather than individuals and whether allocation concealment (if any) was at the cluster level, the individual participant level, or both | Supplementary Material (Randomization) |
| Implementation | | |
| 10a | Who generated the random allocation sequence, who enrolled clusters, and who assigned clusters to interventions | Supplementary Material (Randomization) |
| 10b | Mechanism by which individual participants were included in clusters for the purposes of the trial (such as complete enumeration, random sampling) | Supplementary Material (Randomization) |
| 10c | From whom consent was sought (representatives of the cluster, or individual cluster members, or both) and whether consent was sought before or after randomization | Methods (Ethics) |
| Blinding | | |
| 11a | If done, who was blinded after assignment to interventions (for example, participants, care providers, those assessing outcomes)  and how | Supplementary Material (Masking) |
| 11b | If relevant, description of the similarity of interventions | N/A |
| Statistical Methods | | |
| 12a | Statistical methods used to compare groups for primary and secondary outcomes; How clustering was taken into account | Methods (Statistical analysis) |
| 12b | Methods for additional analyses, such as subgroup analyses and adjusted analyses | Methods (Statistical analyses) |
| **Results** | | |
| Participant Flow | | |
| 13a | For each group, the numbers of participants/clusters who were randomly assigned, received intended treatment, and were analyzed for the primary outcome | Results; Figure 1 |
| 13b | For each group, losses and exclusions after randomization, together with reasons, for both clusters and individual cluster members | Results; Figure 1 |
| Recruitment | | |
| 14a | Dates defining the periods of recruitment and follow-up | Results |
| 14b | Why the trial ended or was stopped | N/A |
| Baseline Data | | |
| 15 | A table showing baseline demographic and clinical characteristics for each group; Baseline characteristics for the individual and cluster levels as applicable for each group | Table 1 |
| Numbers Analyzed | | |
| 16 | For each group, number of participants/clusters (denominator) included in each analysis and whether the analysis was by the original assigned groups | Methods (Statistical analyses); Table S2 |
| Outcomes and Estimation | | |
| 17a | For each primary and secondary outcome, results for each group, and the estimated effect size and its precision (such as 95% confidence interval); Results at the individual and cluster levels as applicable and a coefficient of intracluster correlation (ICC or k) for each primary outcome | Results |
| 17b | For binary outcome, presentation of both absolute and relative effect sizes is recommended | Results |
| Ancillary Analyses | | |
| 18 | Results of any other analyses performed, including subgroup analyses and adjusted analyses, distinguishing pre-specified from exploratory | Results; Supplementary Tables |
| Harms | | |
| 19 | All important harms or unintended effects in each group | N/A |
| **Discussion** | | |
| Limitations | | |
| 20 | Trial limitations, addressing sources of potential bias, imprecision and, if relevant, multiplicity of analyses | Discussion |
| Generalizability | | |
| 21 | Generalizability (external validity, applicability) of the trial findings; Generalizability to clusters and/or individual participants (as relevant) | Discussion |
| Interpretation | | |
| 22 | Interpretation consistent with results, balancing benefits and harms, and considering other relevant evidence | Discussion |
| **Other Information** | | |
| Registration | | |
| 23 | Registration number and name of trial registry | Methods (Registration) |
| Protocol | | |
| 24 | Where the full trial protocol can be accessed, if available | Methods (Registration) |
| Funding | | |
| 25 | Sources of funding and other support (such as supply of drugs), role of funders | Acknowledgements |

**Supplementary Methods**

*Study participants*

Rural subdistricts were selected based on low iron and arsenic levels in the drinking water (to avoid interference with the chlorine-based water treatment intervention) and the absence of major water, sanitation, or focused nutrition programs sponsored by the government or non-governmental organizations during the study period.

Households that planned to move within the following year, that did not own their home, and that drew water from a source with high iron content were excluded. At follow-up, we excluded compounds with no live birth or an index child death.

A data safety monitoring committee convened by icddr,b assessed safety and adverse events.

*Masking*

Study participants, intervention implementers, and outcome assessors were not masked because the interventions delivered visible hardware. One masked laboratory technician (S.D.) conducted the multiplex real-time PCR measurements to detect protozoa infections. Following the pre-registered analysis protocol, two investigators (A.L., A.E.) conducted independent masked data processing and statistical analyses. After replication of all analyses, the results were unmasked.

*Procedures*

The trial recruited and trained local women to serve as community health promoters. Promoters were instructed to visit study compounds in the intervention arms at least once per week during the first 6 months and at least once biweekly throughout the trial (details in Supplementary Material). In actuality, the promoters visited intervention households 5-7 times per month throughout the trial [[1](#_ENREF_1)]. At each visit, community health promoters performed the following activities: resupply intervention products, demonstrate their correct use, provide training on hardware maintenance, resolve hardware problems, and promote intervention usage through discussions, video dramas, storytelling, games, and songs.

Staff distributed sterile fecal collection containers to the primary caregivers of children enrolled in the parasite assessment, instructed them to collect feces from the following morning’s defecation events, and returned to the household on the day of defecation to retrieve the containers. Specimens were transported on ice to the satellite laboratory and stored at -80°C. Specimens were then transported on dry ice to the main icddr,b hospital in Dhaka and analyzed for *Giardia*, *Cryptosporidium*, and *E. histolytica*.

Genomic DNA was extracted from fecal specimens using the QIAamp Fast DNA Stool Mini Kit (QIAGEN, Hilden, Germany) according to the manufacturer’s instructions. 200 mg of stool were used for DNA extraction, and the DNA was eluted in 200 µl of ATE buffer (supplied with the QIAGEN kit).

The protozoa were measured by multiplex real-time PCR using a previously described protocol [[2](#_ENREF_2)]. The assay measures infection prevalence and also allows for the estimation of parasite load by providing a semiquantitative measure of infection intensity – PCR cycle threshold (Ct) values. Briefly, the primers and Taqman probes were designed to amplify the small subunit ribosomal RNA gene for *E. histolytica* (GenBank accession no. X64142) and *Giardia* (GenBank accession no. M54878). The amplified targets for *E. histolytica* and *Giardia* were 134 and 62 base pairs respectively. *Cryptosporidium* primers and Taqman probes were designed to amplify the 151-base pair *Cryptosporidium* oocyst wall protein (COWP; accession no. AF248743). Primers and probes are detailed below and were purchased from Integrated DNA Technologies (Singapore). Amplification reactions were performed in a volume of 25 µL with 12.5 µL of QIAGEN Multiplex PCR Master Mix (QIAGEN, Hilden, Germany). Each reaction contained an additional 2 mM MgCl_2_, 0.4 µM of each Eh-f, Eh-r primers and 0.08 µM Eh-YYT probes for *E. histolytica*, 0.4 µM of each Gd-80F, Gd-127R primers and 0.04 µM of Gd-FT probes for *Giardia*, 1.0 µM of each Cp-583F, Cp-733R primers and 0.2 µM of Cp-TRT probes for *Cryptosporidium*, and 3 µl of the DNA sample. The thermal cycling PCR profile consisted of initial denaturing for 15 min at 95°C followed by 40 cycles of denaturing at 95°C for 20 seconds and annealing at 60°C for 1 minute with fluorescence data collection. Amplification and analysis were performed on the CFX96 Real-Time System (BioRad, Hercules, CA). Each run included three positive controls (*Giardia*, *Cryptosporidium*, and *E. histolytica*) and one negative control. The Ct cutoff for defining a positive sample was <40 [[2](#_ENREF_2)].

*Adjusted analysis*

In a secondary analysis, we estimated adjusted parameters by including variables that were strongly associated with the outcome to potentially improve the precision of our estimates (decrease the SEs); however, we recognize that for binary outcomes gains in precision are very unlikely [[3](#_ENREF_3)]. In accordance with the main study’s analysis plan [[4](#_ENREF_4)] (updated on February 5, 2016, <https://osf.io/63mna/>), we considered and tested the following covariates:

- - Month of measurement, to account for seasonal variation
  - Child age (days)
  - Child sex
  - Child birth order
  - Mother’s age (years)
  - Mother’s height (cm)
  - Mother’s education level (no education, primary, secondary)
  - Household food insecurity (4-level *Household Food Insecurity Access Scale* categories [[5](#_ENREF_5)])
  - Number of children < 18 years in the household
  - Number of individuals living in the compound
  - Distance (in minutes) to the household’s primary drinking water source
  - Housing materials (floor, walls, roof) and household assets

Assets measured: electricity, wardrobe, table, chair or bench, watch or clock, khat, chouki, working radio, working black/white or color television, refrigerator, bicycle (not child’s toy), motorcycle, sewing machine, mobile phone, land phone

Summary of primers and probes used in multiplex real-time PCR assay for detection and differentiation of *E. histolytica*, *G. duodenalis,* and *Cryptosporidium* spp.

| Target organism | Target | Amplicon Size | Accession Number | Name | Oligonucleotide sequence (5’–3’) |
| --- | --- | --- | --- | --- | --- |
| *E. histolytica* | 18s rRNA | 134 bp | X64142 | Eh-f | AAC AGT AAT AGT TTC TTT GGT TAG TAA AA |
|  |  |  |  | Eh-r | CTT AGA ATG TCA TTT CTC AAT TCA T |
|  |  |  |  | Eh-YYT | YYT-ATT AGT ACA AAA TGG CCA ATT CAT TCA-Dark Quencher |
| *G. duodenalis* | 18s rRNA | 62 bp | M54878 | Gd-80F | GACGGCTCAGGACAACGGTT |
|  |  |  |  | Gd-127R | TTGCCAGCGGTGTCCG |
|  |  |  |  | Gd-FT | FAM-CCCGCGGCGGTCCCTGCTAG-DDQ1 |
| *Cryptosporidium* spp. | COWP | 151 bp | AF248743 | Cp-583F | CAA ATT GAT ACC GTT TGT CCT TCT G |
|  |  |  |  | Cp-733R | GGC ATG TCG ATT CTA ATT CAG CT |
|  |  |  |  | Cp-TRT | Texas Red-TGC CAT ACA TTG TTG TCC TGA CAA ATT GAA T-DDQ2 |

*Sample size*

The WASH Benefits study was designed and sized to detect intervention impacts on child length and diarrhea [[6](#_ENREF_6)]. The targeted sample size for the study was 5040 pregnant women in 720 clusters (7 mothers/cluster). Assuming 50% prevalence in the control arm, a village intraclass correlation (ICC) of 0.14, two children measured per enrolled mother, a two-sided alpha of 5%, and 71% successful stool collection and analysis (10/14 samples per cluster), the study had 80% power to detect a relative reduction of 18% in infection prevalence of any protozoan parasite with a single, post-intervention measure.

*Statistical analysis*

We recorded the rates of loss to follow-up and the recovery rates of fecal specimens. To determine whether specimens were missing at random, we compared rates of unavailable fecal specimens across study arms and also the enrolment characteristics of those with unavailable versus available specimens. We also compared the enrollment covariates between study arms at follow-up. We used inverse probability of censoring weighting (IPCW) with TMLE to correct for potential bias due to informative censoring (additional details provided in the pre-registered analysis protocol) [[7](#_ENREF_7)].

*Role of the funding source*

The funder approved the study design, but was not involved in data collection, analysis, interpretation or any decisions related to publication. The corresponding author had full access to all study data and ensured the integrity of the data and the accuracy of the data analysis. The corresponding author had final responsibility for the decision to submit for publication.

**Supplementary References**

1. Parvez S, Azad R, Rahman M, et al. Achieving optimal technology and behavioral uptake of single and combined interventions of water, sanitation, hygiene and nutrition, in an efficacy trial (WASH Benefits) in rural Bangladesh. *Trials*, **2017**; (in review).

2. Haque R, Roy S, Siddique A, et al. Multiplex real-time PCR assay for detection of *Entamoeba histolytica*, *Giardia intestinalis*, and *Cryptosporidium* spp. *Am J Trop Med Hyg*, **2007**; 76(4): 713-717.

3. Pocock SJ, Assmann SE, Enos LE, Kasten LE. Subgroup analysis, covariate adjustment and baseline comparisons in clinical trial reporting: current practice and problems. *Stat Med*, **2002**; 21(19): 2917-2930.

4. Arnold BF, Null C, Luby SP, et al. Cluster-randomised controlled trials of individual and combined water, sanitation, hygiene and nutritional interventions in rural Bangladesh and Kenya: the WASH Benefits study design and rationale. *BMJ Open*, **2013**; 3(8): e003476.

5. Coates J, Swindale A, Bilinsky P. Household Food Insecurity Access Scale (HFIAS) for Measurement of Food Access: Indicator Guide. Washington, DC: Academy for Educational Development, Food and Nutrition Technical Assistance Project (FANTA): 2007.

6. Arnold BF, Galiani S, Ram PK, et al. Optimal recall period for caregiver-reported illness in risk factor and intervention studies: a multicountry study. *Am J Epidemiol*, **2013**; 177(4): 361-370.

7. Little RJ, D'Agostino R, Cohen ML, et al. The prevention and treatment of missing data in clinical trials. *N Engl J Med*, **2012**; 367(14): 1355-1360.
